# Tin-Substituted Chalcopyrite: An *n*-Type Sulfide with Enhanced Thermoelectric Performance

**DOI:** 10.1021/acs.chemmater.2c00637

**Published:** 2022-06-25

**Authors:** Sahil Tippireddy, Feridoon Azough, Vikram, Frances Towers Tompkins, Animesh Bhui, Robert Freer, Ricardo Grau-Crespo, Kanishka Biswas, Paz Vaqueiro, Anthony V. Powell

**Affiliations:** † Department of Chemistry, University of Reading, Whiteknights, Reading RG6 6DX, United Kingdom; ‡ Department of Materials, University of Manchester, Manchester, M13 9PL, United Kingdom; § New Chemistry Unit, Jawaharlal Nehru Centre for Advanced Scientific Research, Jakkur, Bangalore 560064, India

## Abstract

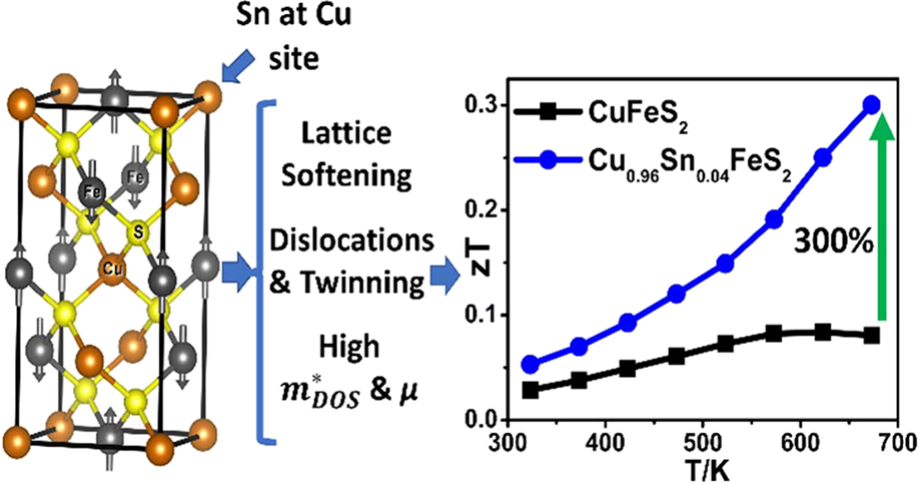

The dearth of *n*-type sulfides with thermoelectric performance comparable to that of their *p-*type analogues presents a problem in the fabrication of all-sulfide devices. Chalcopyrite (CuFeS_2_) offers a rare example of an *n*-type sulfide. Chemical substitution has been used to enhance the thermoelectric performance of chalcopyrite through preparation of Cu_1-*x*_Sn_*x*_FeS_2_ (0 ≤ *x* ≤ 0.1). Substitution induces a high level of mass and strain field fluctuation, leading to lattice softening and enhanced point-defect scattering. Together with dislocations and twinning identified by transmission electron microscopy, this provides a mechanism for scattering phonons with a wide range of mean free paths. Substituted materials retain a large density-of-states effective mass and, hence, a high Seebeck coefficient. Combined with a high charge-carrier mobility and, thus, high electrical conductivity, a 3-fold improvement in power factor is achieved. Density functional theory (DFT) calculations reveal that substitution leads to the creation of small polarons, involving localized Fe^2+^ states, as confirmed by X-ray photoelectron spectroscopy. Small polaron formation limits the increase in carrier concentration to values that are lower than expected on electron-counting grounds. An improved power factor, coupled with substantial reductions (up to 40%) in lattice thermal conductivity, increases the maximum figure-of-merit by 300%, to *zT* ≈ 0.3 at 673 K for Cu_0.96_Sn_0.04_FeS_2_.

## 1 Introduction

Thermoelectric (TE) materials offer the ability to convert waste heat into useful electrical energy and are promising for a range of applications in the automotive, aerospace, manufacturing, energy, and electronic industries.^1^ The energy conversion efficiency of a thermoelectric material depends on a dimensionless quantity, the figure-of-merit, *zT = S^2^*σ*T/*κ. For a high *zT*, the material should possess a high electrical conductivity (*σ*) and Seebeck coefficient (*S*), together with a low thermal conductivity (κ). The thermal conductivity comprises electronic (κ_e_) and lattice (κ_L_) components. However, obtaining a high *zT* is challenging due to the conflicting dependence of *S*, σ, and κ_e_ on the charge-carrier concentration. Using approaches such as doping, nanostructuring, band-structure engineering, and nanocompositing,^2,3^ high figures-of-merit have been achieved in many state-of-the-art thermoelectric materials including PbTe,^4,5^ SnSe,^6,7^ and Bi_2_Te_3_.^8,9^ However, these materials contain relatively toxic, expensive, and/or rare elements. The search for alternative materials containing relatively abundant and cheap elements has led to the identification of ternary^10−19^ and quaternary^20−23^ sulfides, some of which also exist as natural minerals, as promising candidates. However, while this has led to significant improvements in the figure-of-merit of *p-*type sulfides, there continues to be a dearth of *n*-type analogues with comparable performance.^24^

**Figure 1:**
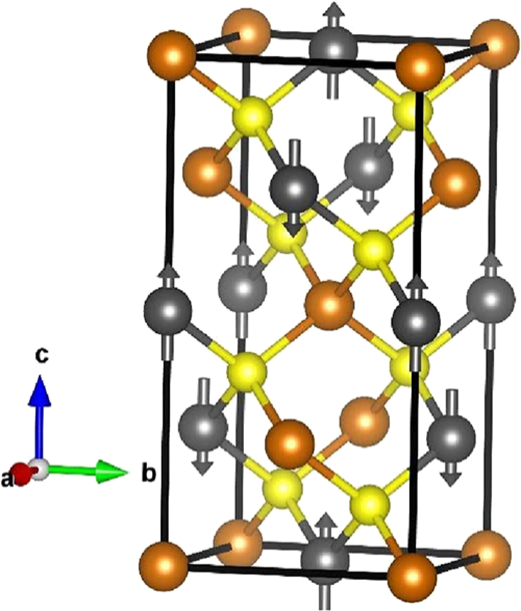
The tetragonal (*I*4̅2*d*) crystal structure of CuFeS_2_ where Cu, Fe, and S are denoted by orange, gray, and yellow balls, respectively. The arrows indicate the direction of the individual atomic moments associated with the iron cations, illustrating the anti-parallel arrangement of moments in adjacent layers of iron cations.

Among the mineral-related sulfides, chalcopyrite, CuFeS_2_, has been explored as a potential *n*-type candidate for thermoelectric applications in the mid-temperature (400 ≤ *T*/K ≤ 673) range. CuFeS_2_ crystallizes in a tetragonal structure with space group: *I*4̅2*d* (Figure 1), which can be considered as a superstructure of the cubic zinc-blende structure, where zinc is replaced by an ordered arrangement of copper and iron cations that occupy tetrahedral 4*a* and 4*b* sites, respectively.^25^ It exhibits an antiferromagnetic ground state (*T*_N_ = 823 K)^26,27^ in which the moments of iron cations in consecutive layers in the *c*-direction are aligned in an anti-parallel manner (Figure 1). Pristine CuFeS_2_ possesses a high Seebeck coefficient (*S* ≈ −380 to −480 μV K^–1^ at 300 K) as well as a relatively high electrical resistivity (ρ ≈ 0.25–0.4 mΩ m at this temperature),^11,28−30^ resulting in only a moderate power factor. Moreover, CuFeS_2_ exhibits a high thermal conductivity (κ ≈ 7 to 9 W m^–1^ K^–1^ at 300 K).^11,28−31^ Efforts to improve electrical properties have focused on the substitution of Cu^+^ with higher-oxidation state transition-metal cations, including those of Mn, Co, Ni, Zn, Cd, and Pd,^11,12,30,32^ to adjust the carrier concentration and optimize the power factor. The introduction of substituents has also been found to be effective in reducing the lattice thermal conductivity, leading to an improvement in thermoelectric performance.^11,12,30,32^ Transition-metal substitution leads to a maximum figure-of-merit *zT* = 0.45 at 723 K^33^ for Cu_0.88_Ag_0.12_FeS_2_, the highest achieved in sulfur-based chalcopyrite-related materials, while comparable performance has been reported for Cu_0.92_Cd_0.08_FeS_2_ (*zT* = 0.39 at 723 K).^12^ However, Cd is toxic whereas Ag is expensive, thereby making these compositions unsuitable for large-scale production and applications.

Using density functional theory (DFT) calculations for CuFeS_2_, Park et al.^34^ predicted that an optimum charge-carrier concentration of *n* ≈ 6 ***×*** 10^21^ to 8 ***×*** 10^21^ cm^–3^ should lead to a high power factor (≈1 mW m^–1^ K^–2^) and, thus, a high figure-of-merit (∼0.8 at 700 K for an average grain size of ∼20 nm). However, CuFeS_2_ typically has a charge-carrier concentration *ca*. 10^19^ cm^–3^, two orders of magnitude lower than the optimum value predicted in that study. In an effort to increase the charge-carrier concentration substantially and more closely approach this optimum carrier concentration, we have substituted copper with tin through the preparation of a series of materials with the general formula Cu_1-*x*_Sn_*x*_FeS_2_ (0.0 ≤ *x* ≤ 0.1). It has been reported that tin is present in the 4+ oxidation state in ternary and quaternary sulfides including Cu_2_SnS_3_, Cu_4_Sn_7_S_16_, Cu_2_ZnSnS_4_, and Cu_2_FeSnS_4_.^35−38^ Therefore, the substitution of Cu^+^ with Sn^4+^ potentially introduces more electrons per substituent than in the case of the previously-explored transition-metal substituents with a 2+ oxidation state. Moreover, tin is a relatively cheap and non-toxic element, thus making it a better candidate for large-scale thermoelectric applications. Our results demonstrate that while the 4+ substitution increases the charge-carrier concentration, the increase is lower than expected. Nevertheless, an optimized carrier concentration combined with a high density-of-states effective mass results in a significant increase in the power factor. The substitution also results in lattice softening and enhanced phonon scattering due to lattice strain and generation of point defects. The resulting reduction in thermal conductivity, coupled with improvements in the power factor, leads to an enhanced figure-of-merit at low levels of tin substitution. The comprehensive analysis of electronic and thermal transport properties of Cu_1-*x*_Sn_*x*_FeS_2_ using a combination of theoretical calculations and experimental data that is reported here suggests an effective strategy to improve the thermoelectric properties of CuFeS_2_.

## 2 Methods

Materials of the general formula, Cu_1-*x*_Sn_*x*_FeS_2_ (0.0 ≤ *x* ≤ 0.1) were prepared by solid-state synthesis and consolidated by hot pressing. Mixtures of appropriate stoichiometric amounts of Cu (Sigma-Aldrich, 99.5%), Fe (Alfa Aesar, 99%+), and Sn (Alfa Aesar, 99.8%) powders, together with S flakes (Sigma-Aldrich, 99.99%) were transferred to fused silica tubes, which were sealed under a vacuum (∼10^–3^ mbar). Reaction mixtures were heated to 723 K and held at this temperature for 150 h before being cooled slowly (0.4 K min^–1^) to room temperature. The products were ground before refiring in an evacuated fused silica tube at 1173 K for 48 h, followed by slow cooling (0.4 K min^–1^) to room temperature. The products from the second firing were ground and hot-pressed at 873 K for 30 min under a pressure of 80 MPa to produce pellets for characterization and thermoelectric measurements. The densities of the pellets measured by the Archimedes method, using an AE Adam PW 184 balance, were >97% of the theoretical value.

Powder X-ray diffraction of the final powdered product and hot-pressed samples was carried out using a Bruker D8 Advance diffractometer (Cu K_α1_: λ = 1.5405 Å), equipped with a LynxEye detector. Powder diffraction data for hot-pressed samples provided no evidence for preferred orientation. Rietveld analysis of the powder diffraction data was performed using FullProf software.^39^ X-ray photoelectron spectroscopy (XPS) was performed with a Kratos Axis spectrometer using Al K_α_ (1486.6 eV) radiation. The spectra were calibrated with C 1s binding energy (B.E. = 284.8 eV), and a Shirley-type background was applied. The peaks were then deconvoluted and fitted with appropriate Lorenz/Gaussian functions using CasaXPS software.

Microstructural investigations were carried out using a SIRION FEI FEG-SEM followed by energy-dispersive X-ray spectroscopy (EDS) elemental point analysis and mapping using a TESCAN MIRA LC FEG (SEM) equipped with an Oxford Instrument SDD energy dispersive detector. Electron backscatter diffraction (EBSD) data were collected using a TESCAN MIRA LC FEG scanning electron microscope (SEM) equipped with an Oxford Instrument SDD EDS detector and an Oxford Instrument Symmetry EBSD detector. Selected-area electron diffraction (SAED) and high-resolution transmission electron microscopy (HRTEM) were performed using an FEI FEGTEM (Tecnai G2, Hillsboro, OR) operating at 300 kV.

The electrical resistivity and Seebeck coefficient on consolidated materials were measured in a direction perpendicular to the pressing direction, over the temperature range 323 ≤ *T*/K ≤ 673 using a Linseis LSR-3 system, while Hall effect measurements were carried out at room temperature using an Ecopia HMS-3000 system. Thermal diffusivity was measured parallel to the pressing direction using a Netzsch LFA-447 NanoFlash instrument (323 ≤ *T*/K ≤ 573) and an Anter Flashline-3000 system (573 ≤ *T*/K ≤ 673 K). The thermal conductivity was determined from the diffusivity data using, κ = α*C*_p_*d*, where α denotes the thermal diffusivity, *C_p_* is the specific heat capacity, and *d* is the sample density. The Dulong–Petit equation was used to calculate the specific heat capacity, *C*_p_ = 0.52 J g^–1^ K^–1^. The uncertainties for electrical resistivity, Seebeck coefficient, and thermal conductivity are 5, 5, and 10%, respectively. Considering the combined uncertainties of all the measurements, the uncertainty in the calculation of figure-of-merit (*zT*) is estimated to be ca. 15%. An Epoch 650 Ultrasonic Flaw Detector (Olympus) with a transducer frequency of 5 MHz was used for the longitudinal (*v*_l_) and transverse (*v*_t_) sound velocity measurements at room temperature using disc-shaped consolidated samples.

All the DFT calculations were carried out using the VASP code^40−42^ with a projected augmented-wave basis^43^ and the generalized gradient approximation (GGA) exchange-correlation functional of Perdew–Burke–Ernzerhof (PBE).^44^ In order to account for the strong correlations of the Fe 3d electrons, the Hubbard correction method (GGA + U) was used with the formulation of Dudarev et al.^45^ implemented in VASP and an *U*_eff_ of = 3 eV. The zero-damping DFT-D3 method of Grimme^46^ was employed to account for long-range dispersion forces, bringing the lattice parameters in close agreement with the experimentally reported values. A converged plane wave cutoff of 385 eV was used in all the calculations. The tetrahedron method with Blöchl corrections^47^ was used to calculate the electronic density of states (DOS). Cell volume, shape and atomic positions for all the structures were fully relaxed using a conjugate gradient algorithm until the forces on each atom fell below 10^–4^ eV Å^–1^. A fully converged automated Γ-centered *k*-point mesh was used to define the Brillouin zone. Spin-polarized calculations were performed to include the Fe magnetic moments with antiferromagnetic ordering in CuFeS_2_ (which was found to be more stable than the ferromagnetic ordering by 0.58 eV per primitive unit cell). The Fe atoms are in a high-spin configuration with a moment of 3.59μ_B_, in close agreement with the experimental value of 3.67μ_B_^27^ determined using neutron diffraction, and with reported theoretical values of 3.62–3.89μ_B_.^34,48,49^ The tetragonal CuFeS_2_ in space group *I*4̅2*d* was simulated using the optimized lattice parameters *a* = *b* = 5.27 Å and *c* = 10.41 Å, which are in good agreement with previous theoretical reports^34^ and the lattice parameters obtained experimentally in the current study. The harmonic and anharmonic force constants were calculated *via* the machine-learning regression algorithms implemented in the hiPhive package^50^ using settings optimized in a previous study^51^ of the thermal conductivities of chalcopyrite-structured semiconductors. The force constants were used to calculate the lattice thermal conductivity using the ShengBTE code.^52^

In the case of Sn-substituted CuFeS_2_, the small polaron formation scenario where neighboring Fe^3+^ is reduced to Fe^2+^ was simulated by modifying the geometric environment around the substitution site to make it suitable to a reduced Fe species.^53^ To do this, we pre-relaxed the configuration after substituting the neighboring Fe atom by an atom that adopts a 2+ state and with a similar ionic radius as Fe^2+^ (we used Zn^2+^), which is then replaced again by Fe, and the configuration is re-relaxed. The reduction of Fe species was then confirmed by Bader charge analysis.^54,55^

## 3 Results and Discussion

**Figure 2:**
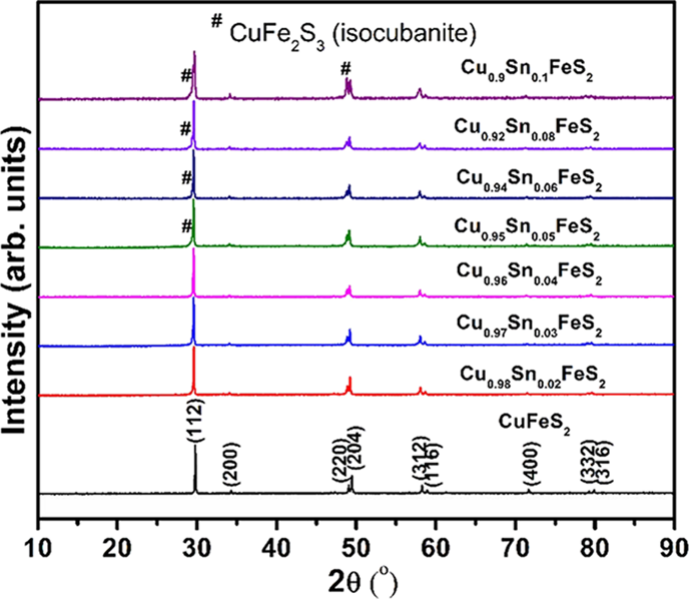
X-ray diffraction patterns of Cu_1-*x*_Sn_*x*_FeS_2_ (0.0 ≤ *x* ≤ 0.1) samples indexed with the chalcopyrite tetragonal phase.

**Figure 3:**
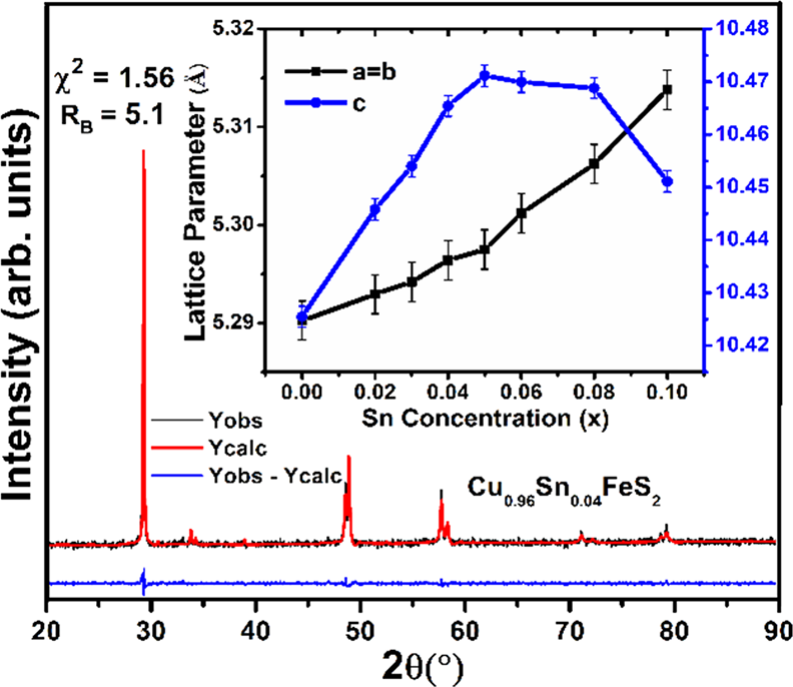
Rietveld analysis of the XRD pattern for Cu_0.96_Sn_0.04_FeS_2_. The inset shows the *a* (=*b*) and *c* lattice parameters as a function of Sn concentration (*x*).

Powder X-ray diffraction data (Figure 2) for Cu_1-*x*_Sn_*x*_FeS_2_ (0 ≤ *x* ≤ 0.1) can be indexed on the basis of a tetragonal unit cell, confirming the presence of a chalcopyrite-related phase throughout the composition range. The low levels of tin incorporation, coupled with the similar X-ray scattering powers of copper and iron, make it impossible to establish unambiguously the location of the incorporated tin cations, and therefore, all structural refinements were performed on the basis that tin substitutes at the copper (4*a*) sites (Figure 3). No reflections arising from secondary phases are observed up to a composition with *x* = 0.04. However, materials with higher tin contents (*x* ≥ 0.05) exhibit reflections assignable to a cubic phase identified as CuFe_2_S_3_ (isocubanite), which adopts a structure consisting of a ccp array of anions with a statistical distribution of Cu and Fe cations on half of the tetrahedral sites (Figure S1a). The intensity of the peaks arising from CuFe_2_S_3_ increases with increasing tin concentration (Figure S1b), with volume fractions determined by Rietveld analysis increasing from 8 vol % at *x* = 0.05 to 32 vol % at *x* = 0.1. The compositional variation of lattice parameters evaluated from Rietveld analysis (Figure 3) reveals that the *a*-lattice parameter increases almost monotonically with increasing tin content, whereas the *c*-parameter saturates at *x* = 0.05 before decreasing at higher tin contents. This suggests a solubility limit of ca. 4–5 at % of tin, with further increases in tin content resulting in the formation of CuFe_2_S_3_.

**Figure 4:**
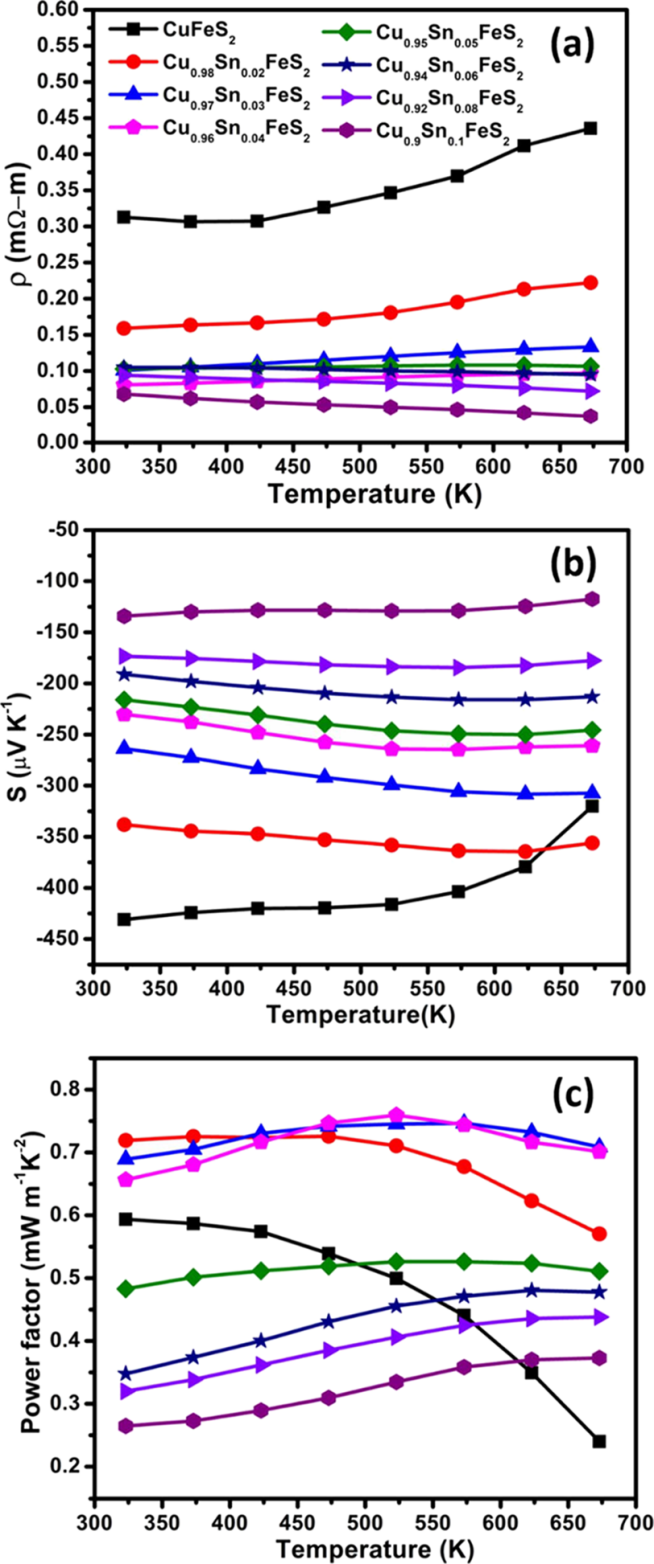
(a) Electrical resistivity (ρ), (b) Seebeck coefficient (*S*), and (c) power factor (PF) of Cu_1-*x*_Sn_*x*_FeS_2_ (0.0 ≤ *x* ≤ 0.1) samples as a function of temperature.

The electrical resistivity (ρ) of unsubstituted CuFeS_2_ (Figure 4a) is ca. 0.31 mΩ m at 323 K and shows a metallic ρ(*T*) dependence. The Seebeck coefficient of CuFeS_2_ is negative (Figure 4b), consistent with electrons as the majority charge carriers and the *n*-type nature of CuFeS_2_. Despite the metallic ρ(*T*) dependence, the Seebeck coefficient is high (*S* = −430 μV/K at 323 K) and almost constant up to 573 K. This value is consistent with previous literature reports. However, as is clear from Table S2, there are wide variations in the electrical-transport properties of CuFeS_2_, with Seebeck coefficients in the range of −320 to −500 μV K^–1^ having been reported. These variations are likely to be associated with slight compositional variations arising from differences in synthesis and processing conditions. The marked decrease in Seebeck coefficient at higher temperatures may be associated with the thermal activation of the minority charge carriers. The low concentration of the minority carriers (holes) results in a low hole contribution to the conductivity, but the hole contribution to the Seebeck coefficient is increased at low carrier contents. The presence of magnetic ions may also influence the *S*(*T*) dependence at higher temperatures as the magnetic ordering transition at ca. 800 K is approached, resulting in significant changes in band structure. The metal-like ρ(*T*) behavior, coupled with a large Seebeck coefficient and high electrical resistivity, indicates degenerate semiconductor behavior and is consistent with previous reports for synthetic CuFeS_2_.^30,32,33,56^

The electrical resistivity falls markedly on the partial replacement of copper with tin. For example, the electrical resistivity at 323 K is reduced by 50% on the introduction of just 2 at. % of tin in Cu_0.98_Sn_0.02_FeS_2_. The electrical resistivity continues to fall at higher tin contents, although the changes become less pronounced. While pristine CuFeS_2_ exhibits a sharp decrease in the Seebeck coefficient at ca. 573 K, in contrast, tin-substitution results in a much weaker *S*(*T*) dependence and appears to decrease the effect of bipolar conduction. For a composition with *x* = 0.02, the Seebeck coefficient falls to ca. −340 μV K^–1^ at 323 K; a 20% reduction from that of pure CuFeS_2_, while the electrical resistivity shows an almost 50% reduction at the same temperature. Hence, low levels of tin substitution (*x* ≤ 0.04) appear to be more effective in reducing the electrical resistivity compared to the Seebeck coefficient, which contributes to relatively high power factors (Figure 4c). Materials with higher levels of tin incorporation contain increasing amounts of the isocubanite (CuFe_2_S_3_) phase as evidenced by X-ray diffraction. Since the electrical resistivity of isocubanite is an order of magnitude lower (ρ ≈ 0.01 mΩ m at 323 ≤ *T*/K ≤ 723)^57,58^ than that of the Cu_1-*x*_Sn_*x*_FeS_2_ phases, it has an increasingly marked impact on electrical transport properties, effectively decreasing the overall electrical resistivity and significantly modifying its temperature dependence.

The end-member CuFeS_2_ exhibits a power factor (PF) *ca*. 0.6 mW m^–1^ K^–2^ at 323 K that falls to 0.24 mW m^–1^ K^–2^ at 673 K, which is primarily due to the decrease in the Seebeck coefficient. At high temperatures, the highest maximum PF ≈ 0.7 mW m^–1^ K^–2^ at 673 K was achieved for the *x* = 0.03 and 0.04 samples. However, the weak *S*(*T*) dependence shown by the tin-substituted materials results in a much flatter temperature dependence of the power factors, while the magnitude of the power factor is increased. For compositions with *x* > 0.05, although the power factor increases slightly with temperature, the values remain low throughout the measured temperature range.

**Figure 5:**
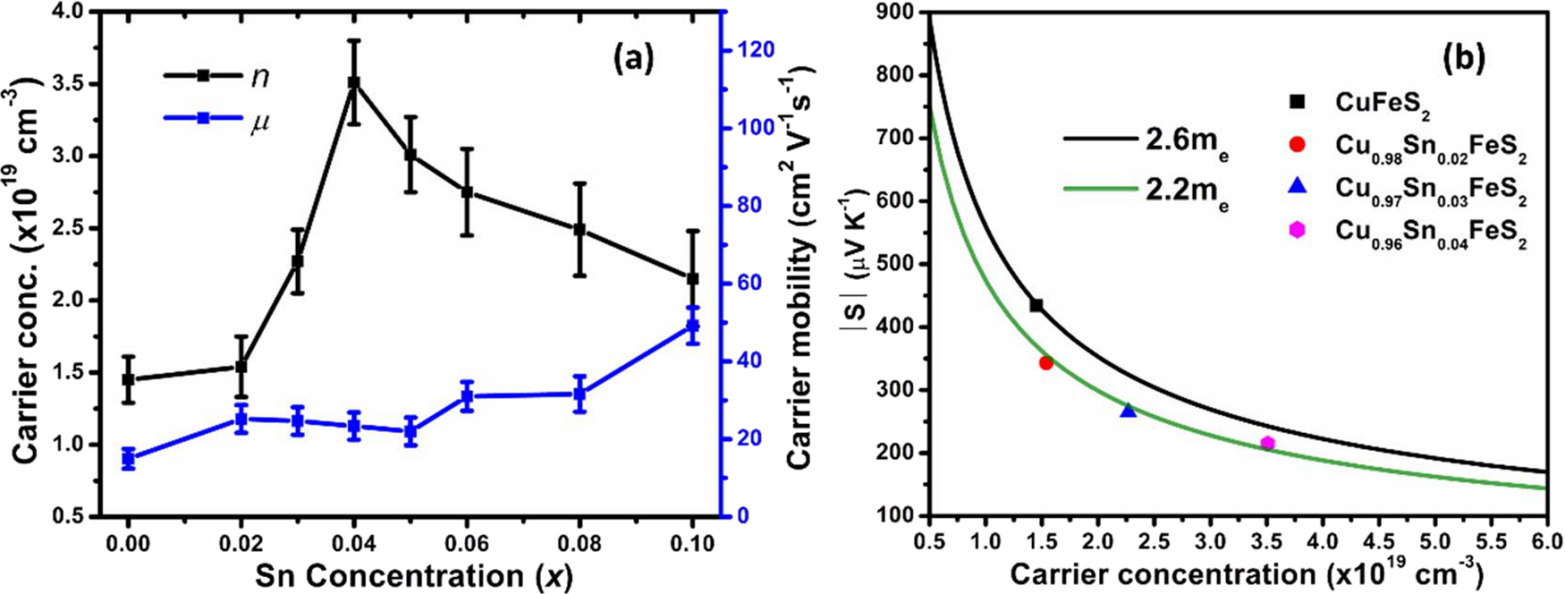
(a) Charge-carrier concentration and mobility of Cu_1-*x*_Sn_*x*_FeS_2_ (0.0 ≤ *x* ≤ 0.1) samples as a function of Sn concentration (*x*). (b) Pisarenko plot (*S* vs *n*) for Cu_1-*x*_Sn_*x*_FeS_2_ (0.0 ≤ *x* ≤ 0.04) samples. A comparison with data for other substituted chalcopyrites is presented in Figure S2.

Room-temperature Hall effect measurements (Table S3) for Cu_1-*x*_Sn_*x*_FeS_2_ (0.0 ≤ *x* ≤ 0.1) presented in Figure 5a reveal that the charge-carrier concentration of the end-member phase, CuFeS_2_, is relatively high (*n* = 1.4(2) × 10^19^ cm^–3^), suggesting the existence of defects in the stoichiometric phase, with a mobility, μ, of 15(3) cm^2^ V^–1^ s^–1^. The mobility shows a marked increase on tin substitution, after which it retains a constant value up to *x* = 0.05, before increasing further at higher levels of substitution. The partial replacement of formally Cu^+^ with Sn^4+^ leads to an increase in the carrier concentration, as expected. However, a maximum carrier concentration of *n* = 3.5(3) × 10^19^ cm^–3^ is reached at Cu_0.96_Sn_0.04_FeS_2_, with the value of carrier concentration decreasing at higher levels of substitution. The maximum carrier concentration achieved was much lower than expected on the basis of electron counting (e.g., ca. 2 × 10^21^ cm^–3^ for each increment of 0.05 in *x*). To the best of our knowledge, the carrier mobility of isocubanite has not been reported. However, the lower reported resistivity and Seebeck coefficient of this phase suggests that the mobility is appreciably higher than that of chalcopyrite: a view that is supported by the DFT calculations of Barbier et al.^57,58^ Therefore, the presence of appreciable amounts of isocubanite at *x* > 0.05 may contribute to the observed marked increase in the charge-carrier mobility at higher levels of tin substitution. Similar increases in mobility on introduction of a higher mobility phase have been reported in nanocomposites of thermoelectrics.^14,59−61^ Carrier scattering due to the presence of a secondary phases becomes significant only when the average mean free path of the carriers is comparable to the dimensions of the secondary phase. DFT calculations^34^ show that the average mean free path of the carriers in CuFeS_2_ is <1 nm, which is much lower than the size of the inclusions of isocubanite (of the order of microns), observed by SEM (Figure S4). Hence, the carrier scattering due to isocubanite is likely to have a small impact on electrical transport properties, given the much higher carrier mobility of this secondary phase.

**Figure 6:**
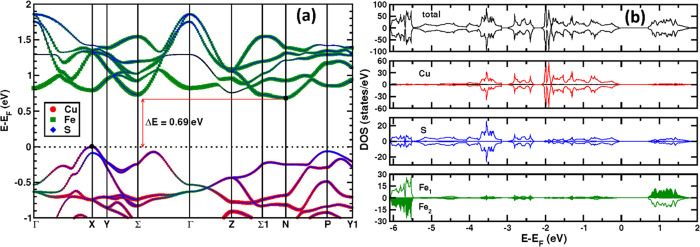
(a) The electronic band structure and (b) the electronic DOS of CuFeS_2_.

DFT simulations have been performed in an effort to understand the electrical transport behavior of CuFeS_2_. Electronic structure calculations (Figure 6a) reveal that the conduction band of CuFeS_2_ consists primarily of contributions from sulfur 3*p* and iron 3*d* states, while the valence band in the region of the Fermi level (*E*_F_) is principally composed of copper 3*d* and sulfur 3*p* states. There is strong hybridization between the Cu *d*-states and S *p*-states as evidenced by the similarity in the partial density of states (DOS) for these states, which is consistent with a strongly covalent Cu–S interaction (Figure 6b). Fe *d*-states are mostly localized as low-lying valence-band states as well as near-edge conduction-band states, indicating a high degree of ionicity in the Fe–S interaction. The *n*-type behavior of CuFeS_2_ identified above implies the presence of intrinsic sulfur vacancies, which serve to move *E*_F_ into the conduction band, and suggests that the band structure in the region of the conduction band edge plays a key role in determining the transport properties of CuFeS_2_. Although there is a clear conduction band minimum (CBM) at the N point, electron pockets exist at the Σ and Z points with energies only 0.04 and 0.06 eV above the CBM, respectively, while the electron pocket at the X point is 0.09 eV higher than the CBM. The close proximity of these electron pockets leads to a high band degeneracy near the CBM. Band degeneracy is known to enhance the Seebeck coefficients even at low carrier concentrations.^62−64^ Combined with the relatively flat nature of the band at the CBM (implying high electron effective mass), this results in the high Seebeck coefficient of pristine CuFeS_2_ (Figure 4b).

DFT calculations also provide insight into the origin of the lower-than-expected increase in carrier concentration on substitution of copper by tin noted above. In particular, the possibility of formation of an Fe^3+^-e^–^ bound state (small polaron), corresponding to a reduction of Fe^3+^ to Fe^2+^, was investigated. Given the limitations of the X-ray diffraction analysis noted above, substitution of tin at both the copper (4*a*) and iron (4*b*) sites was considered. For each mode of substitution, calculations were performed for each of the two possible scenarios: (i) the Fe atom adjacent to the Sn atom is allowed to remain in the Fe^3+^ state (without polaron formation) and (ii) the same Fe atom is “forced” to be in the Fe^2+^ state (with polaron formation). Examination of the ground state energies suggests that in the case of incorporation of Sn at the Cu^+^ site, the polaronic solution is 0.36 eV lower in energy than the non-polaronic solution, i.e., a partial reduction of Fe^3+^ to Fe^2+^ is favored. On the other hand, for Sn substitution at the Fe^3+^ site, the polaronic solution is less stable than the non-polaronic solution by 1.61 eV. This analysis suggests that polaron formation, which results from substitution of tin at the copper site, may be responsible for the lower-than-expected carrier concentration on substitution. Partial reduction of iron, to form a localized Fe^2+^ state effectively reduces the free carrier concentration and leads to a smaller increase in carrier concentration upon tin substitution (at *x* ≤ 0.05) than expected on the basis of replacement of Cu^+^ by Sn^4+^.

Using the charge-carrier concentration (*n*) determined from Hall measurements, Pisarenko plots were constructed for materials with compositions in the range 0.0 ≤ *x* ≤ 0.04 (Figure 5b). Materials with higher tin contents were not analyzed in this way owing to the presence of significant amounts of an impurity phase, as identified from powder X-ray diffraction. The end-member CuFeS_2_ phase exhibits a density-of-states (DOS) effective mass, *m*_d_^*^ = 2.6 *m_e_*, in good agreement with previously reported^30,33,56^ values of *m*_d_^*^ = 2.2 to 2.5 *m*_e_. The high DOS effective mass contributes to the high Seebeck coefficient and is also consistent with the results of DFT calculations discussed above, where the conduction band was shown to contain multiple pockets with a flat band near the conduction band edge. Tin substitution leads to only a slight decrease in the DOS effective mass to *m*_d_^*^ = 2.2 *m*_e_ in Cu_0.98_Sn_0.02_FeS_2_. This relatively high value is maintained at low levels of tin substitution, for compositions in the range 0.0 ≤ *x* ≤ 0.04, while an increased carrier concentration and mobility are also retained. The compositional dependence of the Seebeck coefficient is consistent with the behavior described by the Mott formula
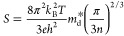
1where *m*_d_^*^, *n*, *e*, *k*_B_, and *h* denote the density-of-states effective mass, charge-carrier concentration, electronic charge, Boltzmann constant, and Planck’s constant, respectively. For compositions in the range 0.02 ≤ *x* ≤ 0.04, the DOS effective mass (numerator) is effectively constant, but the charge-carrier concentration (denominator) increases with increasing tin content, resulting in a decrease of the Seebeck coefficient with *x*. The polaron formation induced by tin substitution reduces the carrier concentration from that expected at a given level of substitution. This enables a relatively high value of the Seebeck coefficient, albeit reduced from that of the end-member phase, to be maintained for levels of tin incorporation of *x* ≤ 0.04. Above this value, the isocubanite impurity, which has a much lower Seebeck coefficient (*S* = −65 to −75 μV K^–1^ at 323 ≤ *T*/K ≤ 723^57,58^ significantly reduces the measured Seebeck coefficient of Cu_1-*x*_Sn_*x*_FeS_2_ (0.05 ≤ *x* ≤ 0.1). Therefore, although it has been predicted^34^ that very high charge- carrier concentrations (ca. 10^21^ cm^–3^) are required for high electrical performance, when combined with a high DOS effective mass and carrier mobility, comparable power factors can be achieved at significantly lower carrier concentrations (ca. 10^19^ cm^–3^). This is in agreement with the suggestion of Park et al.^34^ that high mobility is a more important factor than carrier concentration in optimizing the power factor and, hence, figure-of-merit of chalcopyrite-derived phases. The high mobility affords a better compromise between Seebeck coefficient and electrical conductivity, enabling optimum performance to be realized at lower doping levels.

**Figure 7:**
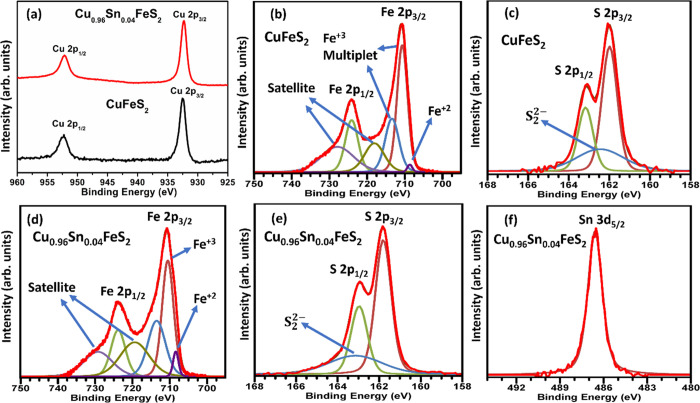
X-ray photoelectron spectroscopy (XPS) spectra of (a) Cu 2p in CuFeS_2_ and Cu_0.96_Sn_0.04_FeS_2_, (b) Fe 2p in CuFeS_2_, (c) S 2p in CuFeS_2_, (d) Fe 2p in Cu_0.96_Sn_0.04_FeS_2_, (e) S 2p in Cu_0.96_Sn_0.04_FeS_2_, and (f) Sn 3d in Cu_0.96_Sn_0.04_FeS_2_.

X-ray photoelectron spectroscopy (XPS) provides support for the presence of iron in mixed oxidation states, resulting from tin substitution, as predicted by the DFT calculations. Data for the end-member phase, CuFeS_2_, and for Cu_0.96_Sn_0.04_FeS_2_ exhibit peaks due to Cu^+^ (Figure 7a), Fe^3+^ (Figure 7b,d), and S^2–^ (Figure 7c,e), consistent with the reported formal oxidation states of CuFeS_2_^11,31,65,66^ (Table S4). The deconvoluted tin XPS spectrum for Cu_0.96_Sn_0.04_FeS_2_ (Figure 7f) exhibits a 3d_5/2_ peak corresponding to Sn^4+^. This supports our assertion that tin is incorporated in the 4+ oxidation state, in common with a number of ternary and quaternary sulfides including Cu_2_SnS_3_, Cu_4_Sn_7_S_16_, Cu_2_FeSnS_4_, and Cu_2_ZnSnS_4_.^35−38^ However, in both spectra, a broad weak feature that can be assigned to the persulfide species, S_2_^2–^, is present, together with a second weak feature associated with Fe^2+^. These peaks may be associated with the presence of trace amounts of a FeS_2_ impurity identified by electron microscopy (*vide infra*). However, the intensity of the peak due to Fe^2+^ in the data from the tin-substituted phase, Cu_0.96_Sn_0.04_FeS_2_, is three times higher than that for the end-member phase, CuFeS_2_. In contrast, the intensity of the persulfide S_2_^2–^ peak in the data for Cu_0.96_Sn_0.04_FeS_2_, is only ca. 75% that in CuFeS_2_. Therefore, our XPS measurements are consistent with the presence of Fe^2+^ in the tin-substituted chalcopyrite phase, as suggested by the DFT calculations, and we propose here that mechanism as the origin of the lower-than-expected increase in carrier concentration on tin substitution.

**Figure 8:**
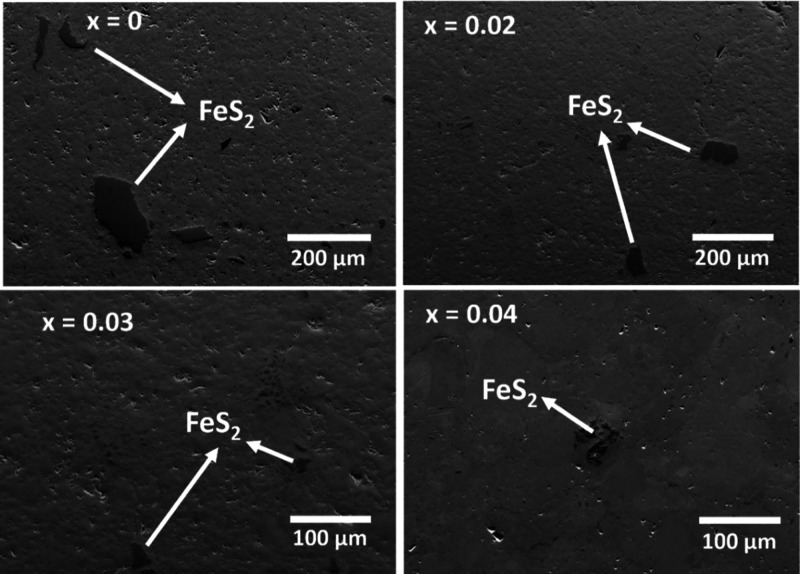
Scanning electron microscopy (SEM) images of Cu_1-*x*_Sn_*x*_FeS_2_ (*x* = 0, 0.02, 0.03, and 0.04) samples, illustrating the reduction of vol % of FeS_2_ phase with an increase in Sn concentration.

Scanning electron microscopy images for Cu_1-*x*_Sn_*x*_FeS_2_ for 0.0 ≤ *x* ≤ 0.04 (Figure 8) and for *x* > 0.04 (Figures S3 and S4) reveal a matrix, identified by EDS as the primary chalcopyrite phase, together with a small amount of a secondary FeS_2_ phase in all compositions. The FeS_2_ impurity can be considered to be the origin of the XPS features assignable to Fe^2+^ and S_2_^2–^ in the end-member phase. At higher magnification (Figure S3), trace amounts of Cu_5_FeS_4_ (bornite) are also discernible in the SEM data, although the formation of this phase is suppressed on substitution by tin. The secondary phases, FeS_2_ and Cu_5_FeS_4_, are present in too low an amount to be detectable by powder X-ray diffraction. For compositions in the range 0.02 ≤ *x* ≤ 0.04, the amount and distribution of the FeS_2_ impurity is reduced substantially compared to that in the end-member (*x* = 0) phase, as was also evidenced in the reduced intensity of the S_2_^2–^ peak in the XPS data for the material with a composition corresponding to *x* = 0.04. This strongly suggests that the increase in the signal from Fe^2+^ in the XPS spectra of the tin-substituted phase is due to the reduction of a fraction of the Fe^3+^ in the main chalcopyrite phase. For compositions with higher tin contents (*x* ≥ 0.05), the SEM data show large amounts of a CuFe_2_S_3_ (isocubanite) type phase (Figure S4), consistent with the powder X-ray diffraction data, in addition to the FeS_2_ secondary phase, while in *x* ≥ 0.08 samples, bornite is also evident at the periphery of the isocubanite phase (Figure S4). Tin substitution has no significant impact on the morphology for compositions of *x* ≤ 0.04 but at higher levels of substitution (*x* ≥ 0.05), a high concentration of microcracks and pores are observed. The compositions of the primary chalcopyrite-type phase in the series Cu_1-*x*_Sn_*x*_FeS_2_ were determined from EDS data (Table S5). The experimentally determined compositions are broadly in line with the nominal compositions. The slight Cu excess may be a consequence of the overlap of the K_α_ and L_α_ characteristic lines of Cu and Fe (from both the main CuFeS_2_ and secondary FeS_2_ phases), while the tin content agrees well with the nominal values.

**Figure 9:**
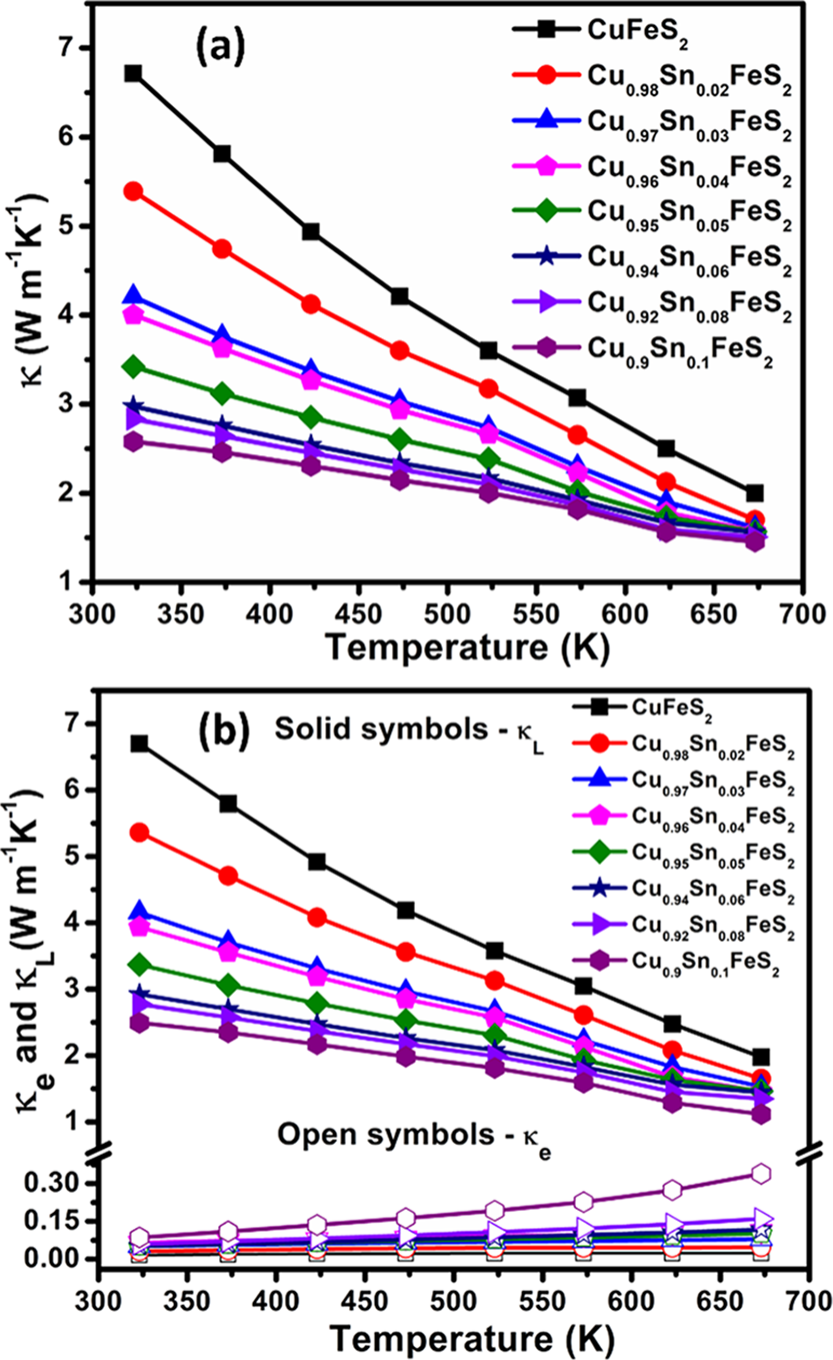
(a) Total (κ), (b) electronic (κ_e_), and lattice (κ_L_) thermal conductivities of Cu_1-*x*_Sn_*x*_FeS_2_ (0.0 ≤ *x* ≤ 0.1) samples as a function of temperature.

The thermal conductivity of the end-member phase, CuFeS_2_, is relatively high (ca. 6.7 W m^–1^ K^–1^ at 323 K), similar to reported values of ∼6.5–8 W m^–1^ K^–1^ (at 323 K),^11,12,30,33,67^ but is substantially reduced on substitution of copper by tin (Figure 9a). The electronic contribution to the thermal conductivity (details of the calculation of κ_e_ are provided in the Supporting Information) is typically *ca*. 1–10% (for 0.0 ≤ *x* ≤ 0.04 at 673 K) of the total thermal conductivity. Therefore, the thermal conductivity of materials in the series Cu_1-*x*_Sn_*x*_FeS_2_ is mainly due to the lattice contribution (κ_L_), and the reduction of 20–40% in the observed thermal conductivity for substituted phases over the range 0.02 ≤ *x* ≤ 0.04, is primarily due to a reduction in κ_L_ (Figure 9b).

Sound velocity measurements (Table 1) were conducted for Cu_1-*x*_Sn_*x*_FeS_2_ with compositions corresponding to *x* = 0, 0.02, and 0.04. In common with previously reported values,^31,67^ the end-member phase, CuFeS_2_, exhibits a high mean sound velocity (Table 1) of *v_m_* = 2831 m s^–1^, accounting for the high measured lattice thermal conductivity, κ_L_. On tin substitution, the mean sound velocity is markedly reduced, which is reflected in the significant reduction of κ_L_ for the tin-substituted materials (Figure 9b). The Debye temperature and elastic modulus, derived from the sound velocity measurements (details of the calculation are provided in the Supporting Information), show corresponding reductions on substitution of copper with tin.

**Table 1:** Various Physical Parameters for Cu_1–*x*_Sn_*x*_FeS_2_ (*x* = 0, 0.02 and 0.04) Samples Showing the Longitudinal (*v*_l_), Transverse (*v*_t_), Average (*v*_avg_), and Mean (*v*_m_) Sound Velocities, Young’s (*E*) and Shear (*G*) Moduli, and Debye Temperature (θ_D_)

sample	*v*_l_ (m/s)	*v*_t_ (m/s)	*v*_avg_ (m/s)	*v*_m_ (m/s)	*E* (Gpa)	*G* (Gpa)	θ_D_ (K)
*x* = 0	4720	2536	3264	2831	69.9	26.94	319
*x* = 0.02	3850	2245	2780	2490	50.59	20.36	280
*x* = 0.04	3500	2150	2600	2372	45.03	18.81	267

**Figure 10:**
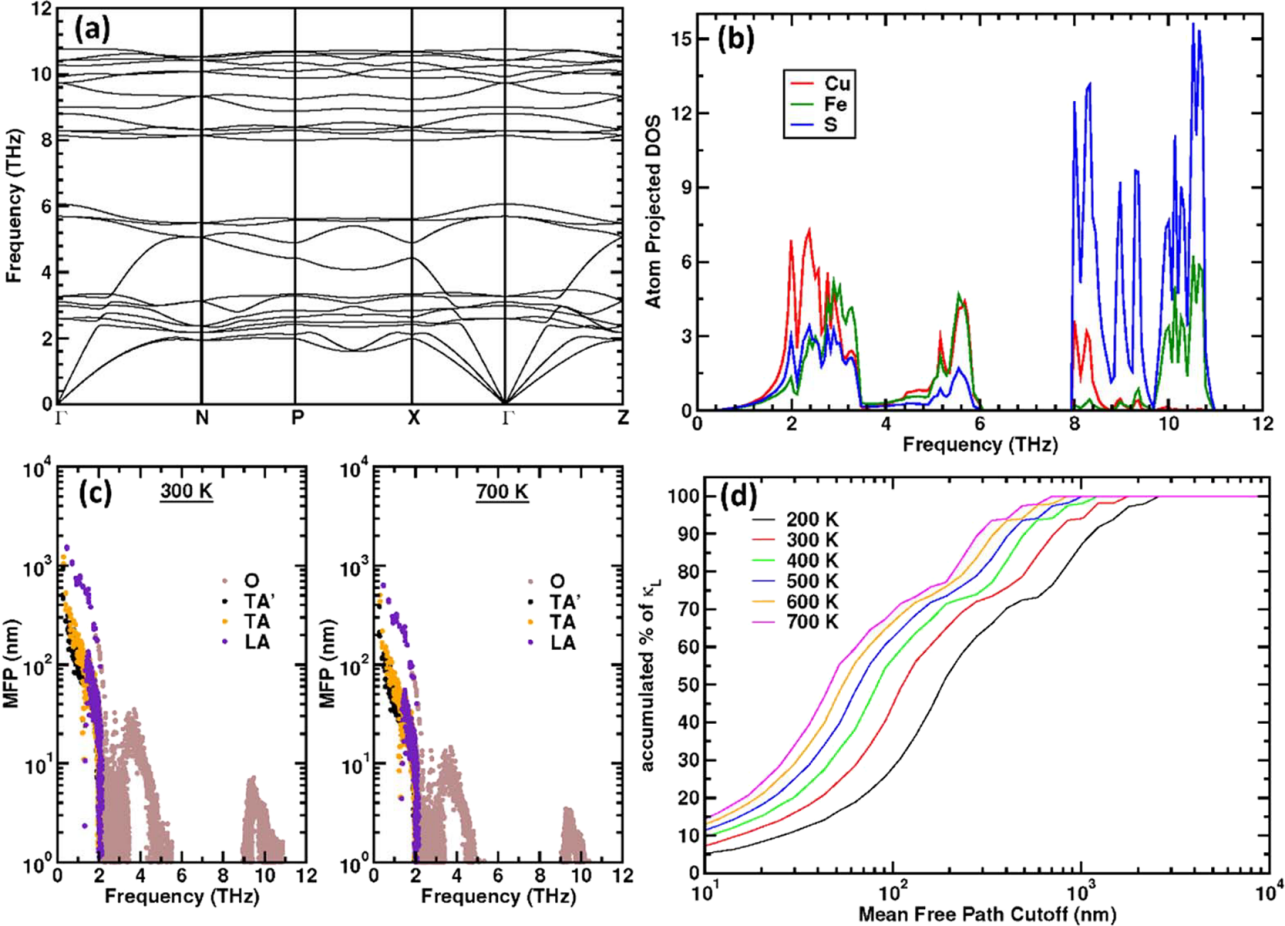
The phonon (a) dispersion curve and (b) DOS of CuFeS_2_. (c) The mean free path (MFP) of optical (O), transverse acoustic (TA) and longitudinal acoustic (LA) phonon modes at 300 and 700 K. (d) The accumulated percentage of lattice thermal conductivity (κ_L_) as a function of mean free path cut-off at different temperatures.

Calculation of the phonon dispersion (Figure 10a) and phonon density-of-states (PDOS) for CuFeS_2_ (Figure 10b) reveal that the majority of the low frequency (<3 THz) heat-carrying acoustic phonon modes lie below 8–12 meV. The average group velocity of these acoustic modes is relatively high (3448 m s^–1^ along Γ – X and 3400 m s^–1^ along Γ – Z) and is responsible for the high mean sound velocity and lattice component of the thermal conductivity. It can be seen from Figure 10b that copper vibrations are the biggest contributor to the acoustic phonons. The low-energy optical modes with frequencies in the range of 4 to 6 THz arise from both Cu and Fe atoms vibrating inside their tetrahedra with almost fixed S positions, whereas the high-frequency (8–11 THz) optical modes are dominated by the motion of S atoms, involving considerable bond stretching.

The frequency dependence of the phonon mean free paths reveal that at 300 K, the majority of the transverse and longitudinal acoustic modes have a mean free path between 50 and 500 nm (Figure 10c). At 700 K, the mean free paths reduce to ∼20–200 nm. We have calculated the effect of nanoparticle and/or grain boundary size on κ_L_ (Figure 10d). This calculation was performed by imposing a cut-off on the mean free path of the phonons. Phonons with a longer mean free path than the cut-off are considered to be scattered, leading to a reduction in κ_L_. At 300 K, a small population of phonons can be scattered effectively for a nanoparticle/grain boundary size of ∼500–600 nm, reducing κ_L_ by ca. 20%. However, the particle size required for a given relative decrease of κ_L_ reduces with increasing temperature. For example, at 700 K, the same 20% reduction would require a nanoparticle/grain boundary size of ∼200 nm. Therefore, in order to achieve the 20 to 40% reduction of κ_L_ at higher temperatures, which is observed experimentally (Figure 9b) for Cu_1-*x*_Sn_*x*_FeS_2_ (0.02 ≤ *x* ≤ 0.04) samples, the nanoparticle/grain boundary size should be <100 nm.

**Figure 11:**
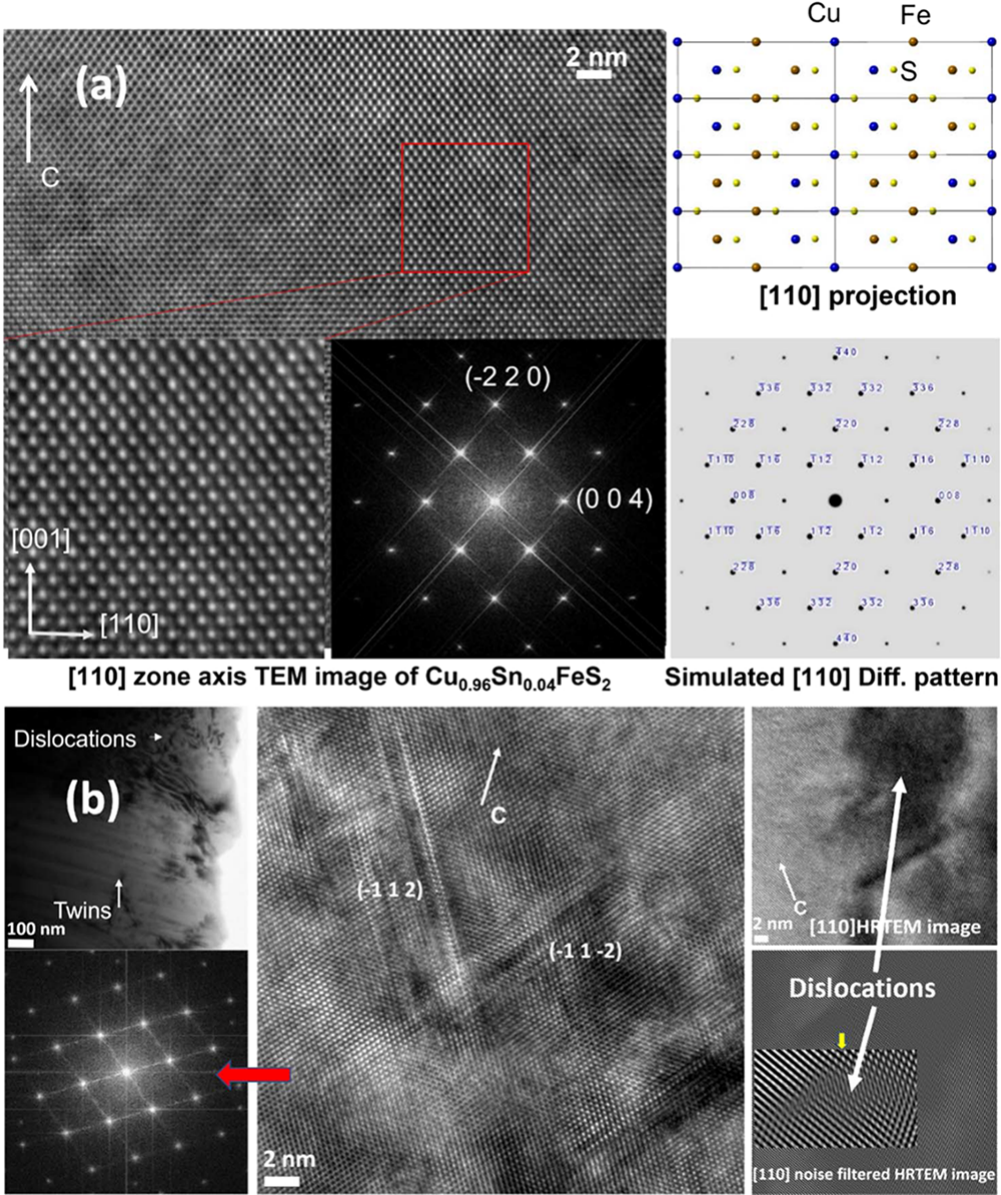
(a) Transmission electron microscopy (TEM) images of the Sn-substituted Cu_0.96_Sn_0.04_FeS_2_ with the selected area electron diffraction (SAED) pattern confirming the tetragonal structure of chalcopyrite. (b) High-resolution TEM images showing dislocations and twinning of the {112} planes.

High-resolution transmission electron microscopy (HRTEM) was used to investigate the micro- and nanostructural features of Cu_0.96_Sn_0.04_FeS_2_ that may have an impact on the mean free path of heat-carrying acoustic phonons. The [110] zone HRTEM image (Figure 11a) of the chalcopyrite phase in Cu_0.96_Sn_0.04_FeS_2_ shows uniform atomic ordering with no short-/long-range disordered domains. The selected area electron diffraction (SEAD) pattern was matched to the simulated diffraction spots of the chalcopyrite structure along the [110] projection. The absence of additional Bragg spots and/or any spot splittings rules out superstructures or lower-symmetry structures, respectively, and confirms that the tetragonal *I*4̅2*d* crystal structure, on which the powder X-ray diffraction data were interpreted, persists into the tin-substituted phases. However, a bright/dark contrast is observed in the HRTEM image (Figure 11b), indicative of dislocations in some grains and grain boundaries. Moreover, a twinning of the {112} plane is observed, as has been found previously in substituted CuFeS_2_ compounds.^32^ Murr et al.^68^ attributed this to the similarity of the tetragonal CuFeS_2_ to the cubic zinc-blende structure in which twinning of the {111} plane is a typical feature and occurs frequently. Therefore, the superstructures of cubic phases (of which chalcopyrite is one example), with the *c*/*a* ratio approaching 2, exhibit similar twinning since the {112} plane of CuFeS_2_ is analogous to the {111} plane of the zinc-blende. In the case of Cu_0.96_Sn_0.04_FeS_2_, *c*/*a* = 1.976, and therefore, twinning is to be expected. These dislocations and twinning features are on the length scale of 10–50 nm and, hence, on the basis of the calculations discussed above, are expected to scatter acoustic phonons. Electron backscattering diffraction (EBSD) data for Cu_0.96_Sn_0.04_FeS_2_ reveals a multiscale grain size distribution (Figure S5) with grain sizes ranging from one to several microns. However, our calculations suggest that since these grains have much larger sizes than the mean free path of the acoustic phonons, they are unlikely to have significant impact in reducing the lattice thermal conductivity.

In order to understand the mechanism behind the reduced lattice thermal conductivity in tin-substituted samples, the Debye–Callaway model^69,70^ was fitted to the experimentally measured thermal conductivities. According to this model, the lattice thermal conductivity can be written as^69,70^
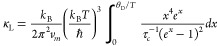
2where *x* = ℏω/*k*_B_*T*, *k_B_* is the Boltzmann’s constant, ℏ is the reduced Plank’s constant, ω is the phonon frequency, *v*_m_ is the mean sound velocity, θ_D_ is the Debye temperature, and τ_c_^–1^ is the total phonon relaxation rate given by
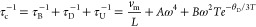
3which considers contributions from grain boundary (τ_B_^–1^), point defect (τ_D_^–1^), and phonon–phonon Umklapp (τ_U_^–1^) scattering processes. In eq 3, *L* is the average grain size, and *A* and *B* are the fitting parameters, which, respectively, are a measure of the contributions of the point defect and Umklapp scattering processes. The fitted parameters (Table 2) reveal that while the Umklapp contribution shows little change with increasing levels of tin substitution, the point defect scattering contribution increases significantly. Since copper cations within the CuS_4_ tetrahedral network are primarily responsible for the heat-carrying acoustic modes (*vide supra*), the substitution by tin, which has a higher mass and larger atomic radius, can lead to significant local disorder and, therefore, point defect scattering of these acoustic modes. The effect of this disorder can be quantified by formulating the coefficient, *A*, in terms of the following expression:
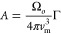
4where, Ω_o_ is the volume of the primitive unit cell and Γ is the scattering parameter. Using the model of Slack^71^ and Abeles,^72^ the scattering parameter Γ can be written as Γ = Γ_M_ + Γ_S_, where Γ_M_ and Γ_S_ are the scattering parameters for mass-difference and strain field fluctuations, respectively, given by
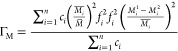
5and,
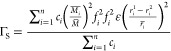
6where , , and . Here, *n* is the number of crystallographic sub-lattices which is 3 for CuFeS_2_ and *c_i_* is the degeneracy of each site in the primitive unit cell (*c*_1_ = *c*_2_ = 1, *c*_3_ = 2 corresponding to Cu, Fe, and S sites, respectively). *M_i_®* and *r_i_®* denote the average mass and radius of the atoms on the *i*^th^ sublattice, respectively. *M*_i_^k^ and *f*_i_^k^ are the atomic mass and fractional occupation of the *k*^th^ atom on the *i*^th^ sublattice, respectively. *M̿* is the total average atomic mass of the compound. Finally, ε is a phenomenological adjustable parameter that was evaluated from fitting eq 2 to the experimental κ_L_*.* For Cu_1-*x*_Sn_*x*_FeS_2_ samples, on the basis of tin substitution occurring at the copper site, as established above, the total scattering parameter can be thus written as
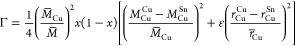
7

**Table 2:** Extracted Scattering Parameters from the Debye–Callaway Model for Cu_1 – *x*_Sn_*x*_FeS_2_ (*x* = 0, 0.02 and 0.04) Samples

sample	*L* (μm)	*A* (× 10^43^ s^–3^)	*B* (× 10^43^ s^–3^)	Γ (× 10^–3^)	Γ_M_ (× 10^–3^)	Γ_S_ (× 10^–3^)	ε
*x* = 0	12.1	2	6				
*x* = 0.02	9.9	10.24	6.2	10.86	7.08	3.78	68
*x* = 0.04	5.4	43.66	6	39.91	13.88	26.03	242

The extracted scattering parameters from substituting the coefficient *A* in eq 2 and fitting the expression to the experimental κ_L_ are shown in Table 2, where it can be seen that both the mass and strain field fluctuation scattering increase with an increase in the tin content. In previous work with transition metals, such as zinc substitution at the copper site,^11^ it was found that only the strain field fluctuation contributes to the reduction in the lattice thermal conductivity because of the similar atomic masses of Zn and Cu. However, tin having a much higher atomic mass (118.71 amu) compared to Cu (63.546 amu) also introduces a considerable contribution from mass-difference fluctuation scattering. Interestingly, at a low level of tin substitution (*x* = 0.02), the scattering is dominated by the mass-fluctuation term (Γ_M_), but for *x* = 0.04, the strain field (Γ_S_) scattering term becomes much larger compared to Γ_M_. Clearly, higher levels of tin substitution induce a significant strain in the lattice.

The reduced elastic moduli and Debye temperature (derived from the sound velocity data shown in Table 1) in the tin-substituted materials compared to CuFeS_2_ further confirm that the local strain fluctuation caused by tin results in a significant lattice softening. Comparing the Γ values of Cu_0.96_Sn_0.04_FeS_2_ determined in the present study with those obtained at similar levels of substitution (2–4 at %) of Zn^11^ and Cd^12^ at the Cu site reveals that the total scattering parameter is much larger in the present case as both Γ_M_ and Γ_S_ have significant contributions. These point defects, combined with a small contribution from the dislocations and twinning faults, observed in the HRTEM data result in effective scattering of phonons over a wide range of the acoustic phonon spectrum. For higher levels of substitution (*x* ≥ 0.05), κ_L_ decreases further through a combination of these effects and the increased amount of impurity phases. The thermal conductivity of isocubanite, identified by powder X-ray diffraction, is somewhat lower than that of chalcopyrite (κ_L_ ≈ 3.6 to 2.5 W m^–1^ K^–1^ over the range 323 ≤ *T*/K ≤ 673)^57,58^ and therefore contributes to the reduction in thermal conductivity of the more highly substituted materials. Moreover, for materials with *x* > 0.05, the microstructure consists of a high concentration of pores and microcracks. The combination of these factors produces strong phonon scattering for compositions with *x* ≥ 0.05 samples, the lowest lattice thermal conductivity (κ_L_ ≈ 1.45 W^–1^ m^–1^ K^–1^ at 673 K) being observed for the *x* = 0.1 composition.

**Figure 12:**
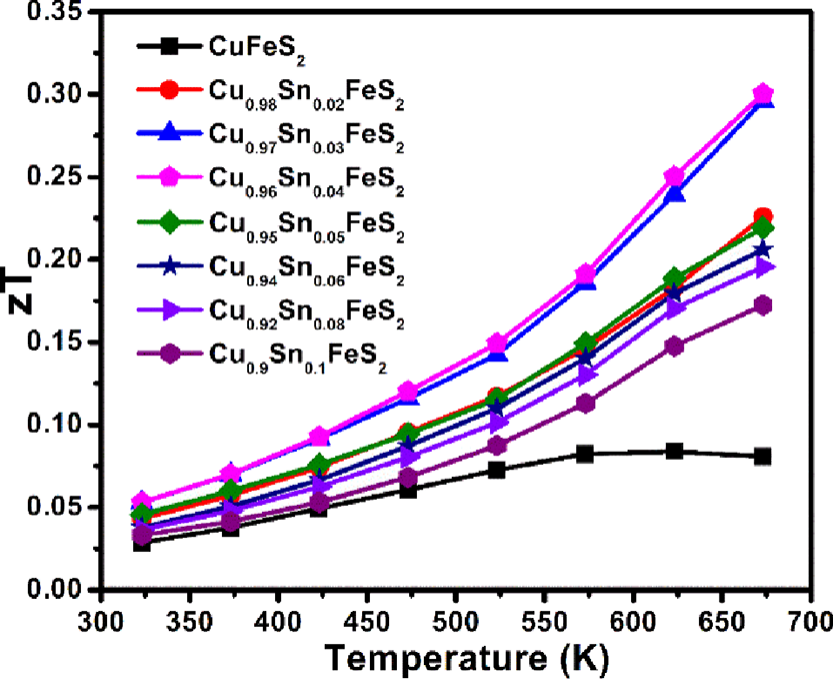
Thermoelectric figure-of-merit (*zT*) of Cu_1-*x*_Sn_*x*_FeS_2_ (0.0 ≤ *x* ≤ 0.1) samples as a function of temperature.

**Figure 13:**
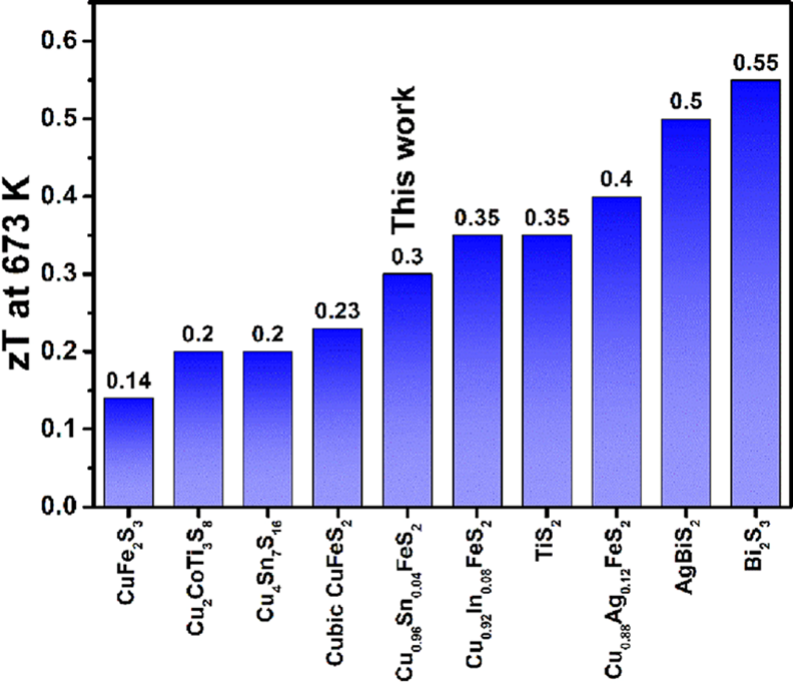
A comparison of the thermoelectric figure-of-merit (*zT*) at 673 K of potential *n*-type sulfide-based materials.^19,33,57,73−78^

The relatively low maximum thermoelectric figure-of-merit (*zT* ≈ 0.08 at 673 K) of the end member-phase CuFeS_2_ (Figure 12) is due to a combination of a high electrical resistivity and a high lattice thermal conductivity. However, the almost 3-fold increase in power factor at low levels of tin substitution, principally due to the combination of an optimum charge-carrier concentration and high DOS effective mass (*m*_d_^*^), coupled with marked reductions in thermal conductivity, leads to a significant increase in the figure-of-merit. The maximum figure-of-merit of *zT* ≈ 0.3 achieved at 673 K in Cu_0.96_Sn_0.04_FeS_2_ is more than 3 times higher than that of the end-member CuFeS_2_ and is larger than that of the majority of the previously reported substituted CuFeS_2_ phases.^11,28,30,32,56^ As noted previously, the thermoelectric performance of *n*-type sulfides is inferior to that of their *p*-type counterparts. Achieving high performance in *n*-type analogues is of paramount importance for the construction of an all-sulfide TE device containing chemically compatible components. The maximum figure-of-merit reported in this work compares favorably with previous reports of *n*-type sulfides (Figure 13). Moreover, the level of performance in the chalcopyrite-derived phase reported here is achieved in a material containing cheap and abundant elements. This offers clear advantages over materials containing rarer elements such as silver, which outweigh the slightly higher performance of the latter.

## 4 Conclusions

The impact on the structural, electronic, and thermoelectric properties of CuFeS_2_ of substitution of copper by tin has been investigated using a range of experimental and computational methods. The solubility limit of Sn was found to be ca. 4 at. %, beyond which an isocubanite secondary phase is observed. While it is not possible to establish the mode of substitution directly by powder X-ray diffraction, the combination of DFT calculations and XPS measurements indirectly support the interpretation that tin preferentially replaces copper at the 4*a* sites. The DFT calculations reveal that substitution of Cu^+^ by Sn^4+^ leads to small polaron formation. This arises from the partial reduction of Fe^3+^ to form localized Fe^2+^ states, which is consistent with the reduction of iron that is observed by XPS. In contrast, DFT predicts that replacement of Fe by Sn would not result in Fe reduction and would therefore be inconsistent with the XPS results.

Polaron formation leads to a lower-than-expected increase in the charge-carrier concentration. Nevertheless, the charge-carrier concentration of CuFeS_2_ is still increased through substitution with tin. While this is reduced from the 3 electrons per substituent that would be expected on the basis of formal charges, the increase in charge-carrier concentration results in a reduction in electrical resistivity. Significantly, this occurs with the retention of a high density-of-states effective mass, which results in a relatively high Seebeck coefficient being maintained. This combination of a low resistivity and high Seebeck coefficient results in a high power-factor (ca. 0.7 mW m^–1^ K^–2^ for Cu_0.96_Sn_0.04_FeS_2_ at 673 K) at modest levels of substitution. This demonstrates the effectiveness of relatively small changes in carrier concentration, similar to that reported for a number of chalcogenide systems.^33,79−81^ Furthermore, the power factor shows a very weak temperature dependence. The nearly flat PF(*T*) response may be an attractive feature for device performance.

Tin substitution also leads to lattice softening and enhanced phonon scattering due to both mass and strain field fluctuations. This, in combination with dislocations and twinning faults, results in a substantial reduction in the lattice thermal conductivity, demonstrating the effectiveness of tin substitution as a promising strategy to increase the phonon scattering across different length scales and improve the thermoelectric performance. On the basis of the calculated accumulated percentage of lattice thermal conductivity (κ_L_) as a function of mean free path (Figure 10d), increasing phonon scattering, particularly for those with mean free paths in the range 50–500 nm, is a key element in the design of new high-performance *n*-type chalcopyrite materials. In the present work, we have shown that the reductions in thermal conductivity achieved through lattice softening, together with the high power factor, results in a maximum figure-of-merit *zT* = 0.3 at 673 K for Cu_0.96_Sn_0.04_FeS_2_. This represents a more than 3-fold increase over that of the parent phase, suggesting that Cu_1-*x*_Sn_*x*_FeS_2_ materials may indeed hold promise as candidates for thermoelectric applications in the mid-temperature range.

## Supporting Information Available

Crystal structure of isocubanite secondary phase and XRD patterns of Cu_1-*x*_Sn_*x*_FeS_2_ (*x* = 0.05–0.1) showing an increase in isocubanite peaks with *x*; comparison of the Seebeck coefficient of pristine CuFeS_2_ with that of the literature; lattice parameters and room-temperature Hall measurement data of Cu_1-*x*_Sn_*x*_FeS_2_ (0.0*≤ x* ≤ 0.1); Pisarenko plots of Sn substituted Cu_1-*x*_Sn_*x*_FeS_2_ (0.0 ≤ *x* ≤ 0.04) samples (present work) and that of other substituted CuFeS_2_ phases in the literature; XPS binding energies and corresponding oxidation states of individual elements in pristine CuFeS_2_ and Sn-substituted Cu_0.96_Sn_0.04_FeS_2_; SEM images of Cu_1-*x*_Sn_*x*_FeS_2_ (0.0 ≤ *x* ≤ 0.1) samples showing the various secondary phases; EDS compositions of the main phase in all the samples; EBSD results for Cu_0.96_Sn_0.04_FeS_2_; calculation details of the Lorenz number, mean and average sound velocities, shear and Young’s moduli, and Debye temperature (PDF)
