# Supplementary material for: Tin-Substituted Chalcopyrite: An n-Type Sulfide with Enhanced Thermoelectric Performance
Source: Chem Mater. 2022 Jun 25;34(13):5860–73. doi: 10.1021/acs.chemmater.2c00637 (PMC9281371; doi:10.1021/acs.chemmater.2c00637)
Supplement: Supplementary file 1 — cm2c00637_si_001.pdf [file cm2c00637_si_001.pdf]

Supporting Information

Tin-Substituted Chalcopyrite: an *n*-Type Sulfide with Enhanced Thermoelectric Performance

Sahil Tippireddy[1], Feridoon Azough[2], Vikram[1], Frances Towers Tompkins[1], Animesh Bhui[3], Robert Freer[2], Ricardo Grau-Crespo[1], Kanishka Biswas[3], Paz Vaqueiro[1], and Anthony V. Powell[1]*

[1]Department of Chemistry, University of Reading, Whiteknights, Reading, RG6 6DX, United Kingdom.

[2]Department of Materials, University of Manchester, Manchester, M13 9PL, United Kingdom.

[3]New Chemistry Unit, Jawaharlal Nehru Centre for Advanced Scientific Research, Jakkur, Bangalore-560064, India.

*Corresponding author email: a.v.powell@reading.ac.uk

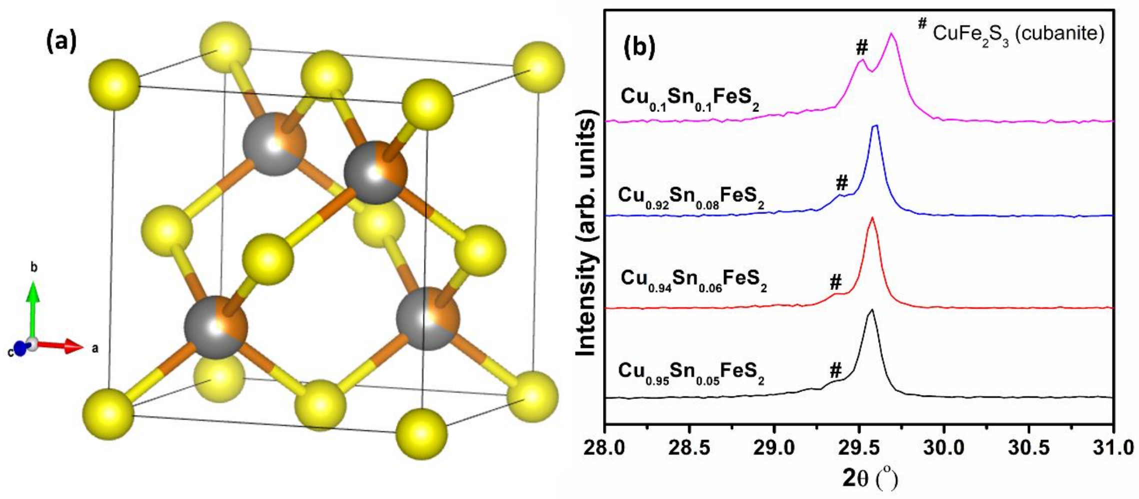

Figure S1. (a) The cubic ($F\bar{4}3m$) crystal structure of $CuFe_2S_3$ isocubanite secondary phase where Cu, Fe and S are denoted by orange, grey and yellow balls, respectively. (b) XRD showing the evolution of isocubanite secondary phase in $Cu_{1-x}Sn_xFeS_2$ (x = 0.05 – 0.1). The isocubanite peak at $2\theta \approx$ 29.3-29.4 increases in intensity with an increase in Sn substitution.

Table S1. Lattice parameters of $Cu_{1-x}Sn_xFeS_2$ (x = 0 – 0.1) described in the space group $I\bar{4}2d$.

Sample	*a*/Å	*c*/Å
$CuFeS_2$	5.2902(4)	10.4254(6)
$Cu_{0.98}Sn_{0.02}FeS_2$	5.2929(4)	10.4458(5)
$Cu_{0.97}Sn_{0.03}FeS_2$	5.2942(3)	10.4541(3)
$Cu_{0.96}Sn_{0.04}FeS_2$	5.2964(5)	10.4654(2)
$Cu_{0.95}Sn_{0.05}FeS_2$	5.2975(2)	10.4711(2)
$Cu_{0.94}Sn_{0.06}FeS_2$	5.3011(7)	10.4699(3)
$Cu_{0.92}Sn_{0.08}FeS_2$	5.3062(2)	10.4688(2)
$Cu_{0.9}Sn_{0.1}FeS_2$	5.3138(3)	10.4511(2)

Table S2. A comparison of the Seebeck coefficient of unsubstituted $CuFeS_2$ at 323 K with literature.

**Seebeck coefficient ($\mu V\ K^{-1}$)**	**Reference**
-430	This work
-320	1
-360	2
-365	3,4
-373	5
-422	6
-450	7,8
-480	9
-500	10,11

Table S3. Room temperature Hall measurement data of $Cu_{1-x}Sn_xFeS_2$ (x = 0 – 0.1) samples.

**Sample**	**Charge carrier concentration ($\times 10^{19}$ cm^{-3})**	**Charge carrier mobility (cm^2 V^{-1} s^{-1})**
$CuFeS_2$	1.4(2)	15(3)
$Cu_{0.98}Sn_{0.02}FeS_2$	1.5(2)	25(4)
$Cu_{0.97}Sn_{0.03}FeS_2$	2.3(2)	25(4)
$Cu_{0.96}Sn_{0.04}FeS_2$	3.5(3)	23(4)
$Cu_{0.95}Sn_{0.05}FeS_2$	3.0(3)	22(4)
$Cu_{0.94}Sn_{0.06}FeS_2$	2.7(3)	31(4)
$Cu_{0.92}Sn_{0.08}FeS_2$	2.5(3)	32(5)
$Cu_{0.9}Sn_{0.1}FeS_2$	2.2(3)	49(5)

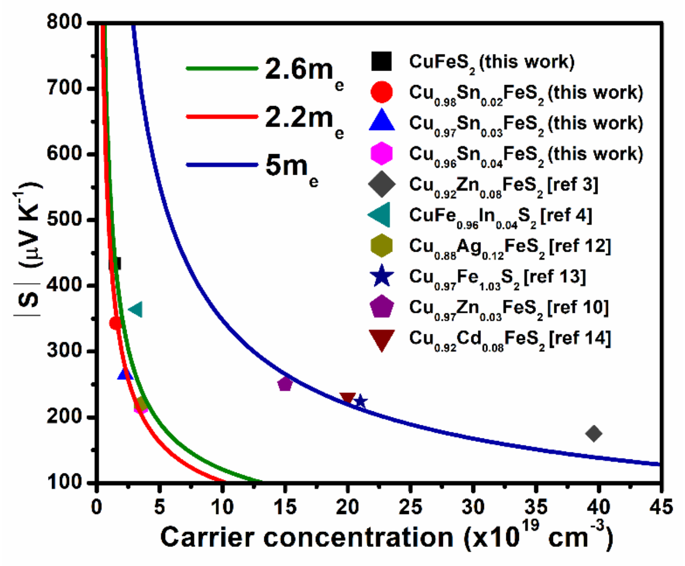

Figure S2. Pisarenko plots of Sn substituted $Cu_{1-x}Sn_xFeS_2$ (x = 0 – 0.04) samples (present work) and that of other substituted $CuFeS_2$ phases in the literature. Many of the high-performing substituted chalcopyrites possess a high DOS effective mass.

Table S4. XPS binding energies and corresponding oxidation states of individual elements in pristine $CuFeS_2$ and Sn-substituted $Cu_{0.96}Sn_{0.04}FeS_2$

**Sample**	**Element**	**Peak**	**B.E(eV)***	**Oxidation state**
**$CuFeS_2$**	Cu	$2p_{3/2}$	932.3	+1
		$2p_{1/2}$	952.2	+1
	Fe	$2p_{3/2}$	710.6	+3
		$2p_{1/2}$	724.1	+3
		2p	708.6	+2
	S	$2p_{3/2}$	161.9	-2
		$2p_{1/2}$	163.1	-2
	S_2^{2-}	2p	162.4	-2
**$Cu_{0.96}Sn_{0.04}FeS_2$**	Cu	$2p_{3/2}$	932.4	+1
		$2p_{1/2}$	952.3	+1
	Fe	$2p_{3/2}$	710.5	+3
		$2p_{1/2}$	723.8	+3
		2p	708.4	+2
	S	$2p_{3/2}$	161.8	-2
		$2p_{1/2}$	162.9	-2
	S_2^{2-}	2p	163.02	-2
	Sn	$3d_{5/2}$	486.6	+4

*The binding energy of the XPS peaks is indexed from the literature and NIST database.

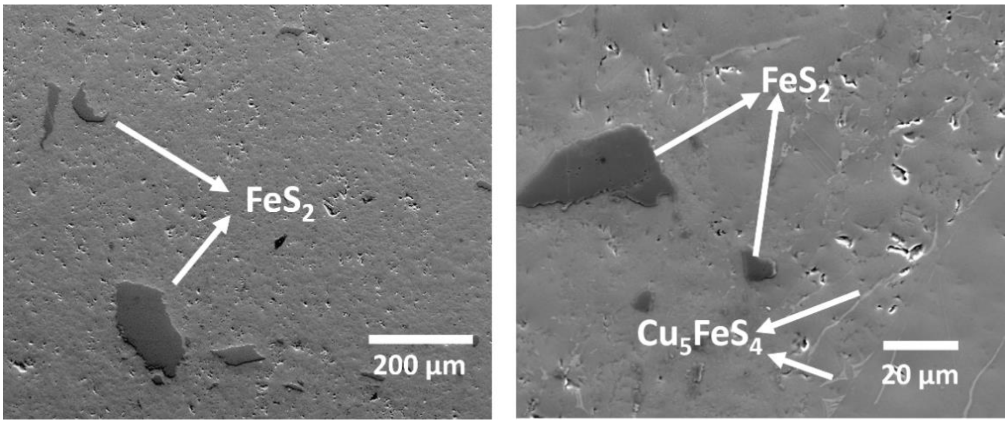

Figure S3. SEM images of pristine $CuFeS_2$ showing the FeS_2 (dark grey) and Cu_5FeS_4 (white) secondary phases.

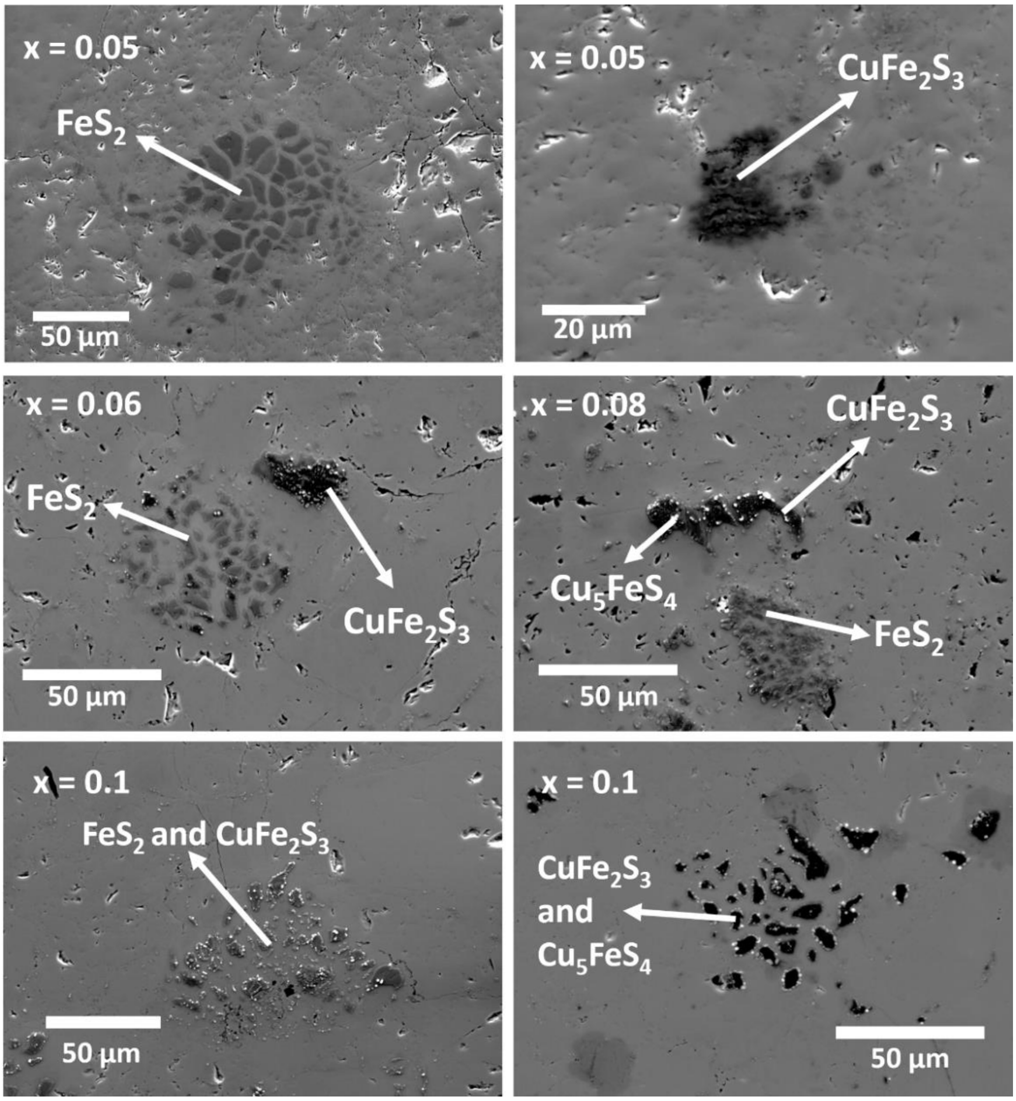

Figure S4. SEM images of $Cu_{1-x}Sn_xFeS_2$ (x = 0.05 – 0.1) samples showing the various secondary phases. For $x \geq 0.06$ samples, the Cu_5FeS_4 bornite phase can be seen as small white spots on the periphery of the $CuFe_2S_3$ (black) isocubanite phase. The x = 0.1 sample contains agglomeration of two secondary phases, unlike other samples.

Table S5. The EDS compositions# of the main phase in all the samples. The secondary phase(s) found from SEM are also listed.

**Nominal composition**	**EDS composition of the main phase***	**Secondary phase(s)**
$CuFeS_2$	$Cu_{1.217\pm0.009}Fe_{1.018\pm0.005}S_2$	FeS_2, Cu_5FeS_4
$Cu_{0.98}Sn_{0.02}FeS_2$	$Cu_{1.19\pm0.003}Sn_{0.019\pm0.002}Fe_{1.002\pm0.005}S_2$	FeS_2
$Cu_{0.97}Sn_{0.03}FeS_2$	$Cu_{1.156\pm0.007}Sn_{0.026\pm0.002}Fe_{0.99\pm0.004}S_2$	FeS_2
$Cu_{0.96}Sn_{0.04}FeS_2$	$Cu_{1.143\pm0.004}Sn_{0.038\pm0.005}Fe_{1.007\pm0.007}S_2$	FeS_2
$Cu_{0.95}Sn_{0.05}FeS_2$	$Cu_{1.127\pm0.02}Sn_{0.047\pm0.003}Fe_{1.005\pm0.013}S_2$	FeS_2, $CuFe_2S_3$
$Cu_{0.94}Sn_{0.06}FeS_2$	$Cu_{1.114\pm0.005}Sn_{0.056\pm0.001}Fe_{1.003\pm0.005}S_2$	FeS_2, $CuFe_2S_3$
$Cu_{0.92}Sn_{0.08}FeS_2$	$Cu_{1.102\pm0.007}Sn_{0.073\pm0.003}Fe_{1.011\pm0.006}S_2$	FeS_2, $CuFe_2S_3$, Cu_5FeS_4
$Cu_{0.9}Sn_{0.1}FeS_2$	$Cu_{0.934\pm0.008}Sn_{0.088\pm0.011}Fe_{1.03\pm0.015}S_2$	FeS_2, $CuFe_2S_3$, Cu_5FeS_4

*Normalized to 2 atoms per unit cell of Sulfur in $CuFeS_2$.

#Due to the overlap of the L_α characteristic lines of Cu and Fe, there may be uncertainties in the quantitative at% from EDS.

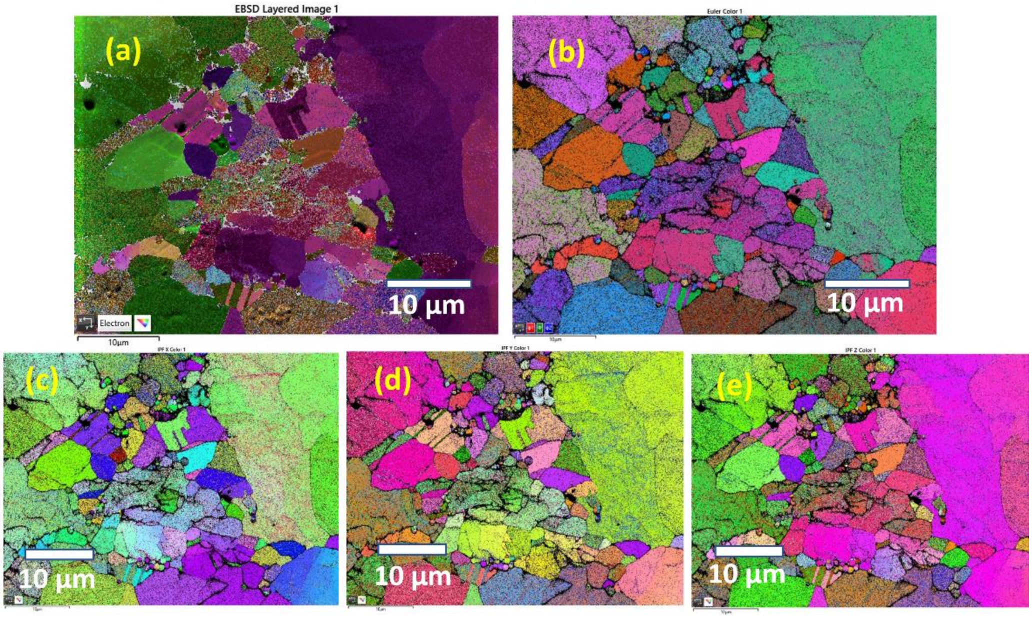

Figure S5. EBSD results for $Cu_{0.96}Sn_{0.04}FeS_2$. (a) grain orientation map. (b) Euler colour map. (c), (d) and (e) represent the x, y, z inverse pole figure maps, respectively.

Calculation Details:

- **Lorenz number**:

The electronic part of the thermal conductivity (κ_e) was calculated from the Wiedemann-Franz relation:

$$\kappa_e = L\sigma T \tag{S1}$$

L is the temperature-dependent Lorenz number and *T* is the temperature. The temperature-dependent Lorenz number was evaluated from the following relation:

$$L = (\frac{k_B}{e})^2 \left(\frac{\left(r+\frac{7}{2}\right)F_{r+5/2}\,(\eta)}{(r+\frac{3}{2})F_{r+1/2}\,(\eta)} - \left[\frac{\left(r+\frac{5}{2}\right)F_{r+3/2}\,(\eta)}{(r+\frac{3}{2})F_{r+1/2}\,(\eta)}\right]^2 \right) \tag{S2}$$

Where k_B is the Boltzmann's constant, η is the reduced Fermi energy that is obtained from Seebeck coefficient values via the relation:

$$S = \pm \frac{k_B}{e} \left(\frac{\left(r+\frac{5}{2}\right)F_{r+3/2}\,(\eta)}{(r+\frac{3}{2})F_{r+1/2}\,(\eta)} - \eta \right) \tag{S3}$$

Here, *F(η)* is the reduced Fermi integral given by:

$$F_n(\eta) = \int_0^\infty \frac{x^n}{1 + e^{x-\eta}}\, dx \tag{S4}$$

And $\eta = E_F/k_BT$ where E_F denotes the Fermi level. Assuming that the main scattering mechanism is acoustic phonon scattering, the value of *r* is taken as -1/2. The Lorenz number at each temperature value is therefore obtained by substituting η and *r* in equation (S2).

- **Mean sound velocity (v_m):**

$$v_m = \left[\frac{1}{3}\left(\frac{2}{v_t^3} + \frac{1}{v_l^3}\right)\right]^{-\frac{1}{3}}$$

and **average sound velocity (v_{avg}):**

$$v_{avg} = (2v_t + v_l)/3$$

where, v_l and v_t are the longitudinal and transverse velocities, respectively.

- **Shear modulus (*G*):**

$$G = dv_t^2$$

Where d and v_t are the density and transverse velocities respectively.

- **Young's modulus (*E*):**

$$E = \frac{dv_t^2\ (3v_l^2 - 4v_t^2)}{(v_l^2 - v_t^2)}$$

- **Debye temperature (θ_D):**

$$\theta_D = \frac{h}{k_B}\left(\frac{3N}{4\pi V}\right)^{1/3} v_m$$

where h is the Plank's constant, k_B is the Boltzmann's constant, N is the number of atoms in the unit cell, V is the volume of the unit cell and v_m is the mean sound velocity.

References:

(1) Lefèvre, R.; Berthebaud, D.; Mychinko, M. Y.; Lebedev, O. I.; Mori, T.; Gascoin, F.; Maignan, A. Thermoelectric Properties of the Chalcopyrite $Cu_{1-x}M_xFeS_{2-y}$ Series (M = Mn, Co, Ni). *RSC Adv.* **2016**, *6*, 55117–55124.

(2) Xie, H.; Su, X.; Hao, S.; Zhang, C.; Zhang, Z.; Liu, W.; Yan, Y.; Wolverton, C.; Tang, X.; Kanatzidis, M. G. Large Thermal Conductivity Drops in the Diamondoid Lattice of $CuFeS_2$ by Discordant Atom Doping. *J. Am. Chem. Soc.* **2019**, *141*, 18900–18909.

(3) Xie, H.; Su, X.; Zheng, G.; Zhu, T.; Yin, K.; Yan, Y.; Uher, C.; Kanatzidis, M. G.; Tang, X. The Role of Zn in Chalcopyrite $CuFeS_2$: Enhanced Thermoelectric Properties of $Cu_{1-x}Zn_xFeS_2$ with In Situ Nanoprecipitates. *Adv. Energy Mater.* **2017**, *7*, 1601299.

(4) Xie, H.; Su, X.; Zheng, G.; Yan, Y.; Liu, W.; Tang, H.; Kanatzidis, M. G.; Uher, C.; Tang, X. Nonmagnetic in Substituted $CuFe_{1-x}In_xS_2$ Solid Solution Thermoelectric. *J. Phys. Chem. C* **2016**, *120*, 27895–27902.

(5) Xie, H.; Su, X.; Yan, Y.; Liu, W.; Chen, L.; Fu, J.; Yang, J.; Uher, C.; Tang, X. Thermoelectric Performance of $CuFeS_{2+2x}$ Composites Prepared by Rapid Thermal

Explosion. *NPG Asia Mater.* **2017**, *9*, 1–12.

(6) Navratil, J.; Kašparová, J.; Plecháček, T.; Beneš, L.; Olmrová-Zmrhalová, Z.; Kucek, V.; Drašar, Č. Thermoelectric and Transport Properties of N-Type Palladium-Doped Chalcopyrite $Cu_{1-x}Pd_xFeS_2$ Compounds. *J. Electron. Mater.* **2019**, *48*, 1795–1804.

(7) Li, J.; Tan, Q.; Li, J. F. Synthesis and Property Evaluation of $CuFeS_{2-x}$ as Earth-Abundant and Environmentally-Friendly Thermoelectric Materials. *J. Alloys Compd.* **2013**, *551*, 143–149.

(8) Carr, W. D.; Morelli, D. T. The Thermoelectric Properties and Solubility Limit of $CuFeS_{2(1-x)}Se_{2x}$. *J. Electron. Mater.* **2016**, *45*, 1346–1350.

(9) Teranishi, T. Magnetic and Electric Properties of Chalcopyrite. *J. Phys. Soc. Japan* **1961**, *16*, 1881–1887.

(10) Tsujii, N.; Mori, T. High Thermoelectric Power Factor in a Carrier-Doped Magnetic Semiconductor $CuFeS_2$. *Appl. Phys. Express* **2013**, *6*, 043001.

(11) Levinský, P.; Hejtmánek, J.; Knížek, K.; Pashchenko, M.; Navrátil, J.; Masschelein, P.; Dutková, E.; Baláž, P. Nanograined n- and p-Type Chalcopyrite $CuFeS_2$ Prepared by Mechanochemical Synthesis and Sintered by SPS. *Acta Phys. Pol. A* **2020**, *137*, 904–907.

(12) Ge, B.; Shi, Z.; Zhou, C.; Hu, J.; Liu, G.; Xia, H.; Xu, J.; Qiao, G. Enhanced Thermoelectric Performance of n-Type Eco-Friendly Material $Cu_{1-x}Ag_xFeS_2$ (x=0–0.14) via bandgap tuning. *J. Alloys Compd.* **2019**, *809*, 151717.

(13) Li. Y.; Zhang, T.; Qin, Y.; Day, T.; Snyder, G. J.; Shi, X.; Chen, L. Thermoelectric Transport Properties of Diamond-Like $Cu_{1-x}Fe_{1+x}S_2$ Tetrahedral Compounds. *J. Appl. Phys.* **2014**, *116*, 203705.

(14) Ge, B.; Hu, J.; Shi, Z.; Wang, H.; Xia, H.; Qiao, G. Integration of Multi-Scale Defects for Optimizing Thermoelectric Properties of n-Type $Cu_{1-x}Cd_xFeS_2$ (x = 0–0.1). *Nanoscale* **2019**, *11*, 17340
